# A deoxynucleoside triphosphate triphosphohydrolase promotes cell cycle progression in *Caulobacter crescentus*

**DOI:** 10.1128/jb.00145-25

**Published:** 2025-06-02

**Authors:** Chandler N. Hellenbrand, David M. Stevenson, Katarzyna A. Gromek, Daniel Amador-Noguez, David M. Hershey

**Affiliations:** 1 Department of Bacteriology, University of Wisconsin-Madison 205263, Madison, Wisconsin, USA; National Institutes of Health, Bethesda, Maryland, USA

**Keywords:** dNTPs, DNA replication, *Caulobacter crescentus*, cell cycle control

## Abstract

Intracellular pools of deoxynucleoside triphosphates (dNTPs) are strictly maintained throughout the cell cycle to ensure accurate and efficient DNA replication. DNA synthesis requires an abundance of dNTPs, but elevated dNTP concentrations in nonreplicating cells delay entry into S phase. Enzymes known as deoxyguanosine triphosphate triphosphohydrolases (Dgts) hydrolyze dNTPs into deoxynucleosides and triphosphates, and we propose that Dgts restrict dNTP concentrations to promote the G1 to S phase transition. We characterized a Dgt from the bacterium *Caulobacter crescentus* termed *flagellar signaling suppressor C* (*fssC*) to clarify the role of Dgts in cell cycle regulation. Deleting *fssC* increases dNTP levels and extends the G1 phase of the cell cycle through a mechanism independent of the response regulator CtrA. Segregation and duplication of the chromosomal origin of replication (*oriC*) are delayed in ∆*fssC*, but the rate of replication elongation is unchanged. We conclude that dNTP hydrolysis by FssC promotes the initiation of DNA replication. This work further establishes Dgts as important regulators of the G1 to S phase transition, and the high conservation of Dgts across all domains of life implies that Dgt-dependent cell cycle control may be widespread in many organisms.

**IMPORTANCE:**

Cells must faithfully replicate their genetic material in order to proliferate. Studying the regulatory pathways that determine when a cell initiates DNA replication is important for understanding fundamental biological processes, and it can also improve the strategies used to treat diseases that affect the cell cycle. Here, we identify a nucleotide signaling pathway that influences when cells begin DNA replication. We show that this pathway promotes the transition from the G1 to the S phase of the cell cycle in the bacterium *Caulobacter crescentus* and propose that this pathway is prevalent in all domains of life.

## INTRODUCTION

All cells proliferate through a highly ordered sequence of events known as the cell cycle. Precise coordination of the cell cycle is critical for survival, as improper control can lead to genome instability or cell death. For instance, the intracellular pools of deoxynucleoside triphosphates (dNTPs) must be strictly regulated throughout the cell cycle. As the precursors of DNA, physiological dNTP levels are crucial for accurate and efficient DNA replication (1). Perturbed dNTP levels can decrease polymerase fidelity, cause DNA damage, and stall replication forks (2–4).

The regulation of dNTP levels is coordinated with DNA synthesis (5). dNTP concentrations increase after the initiation of DNA replication to provide substrates for DNA polymerase. Ribonucleotide reductase (RNR) increases dNTP levels during DNA replication by synthesizing dNTPs from ribonucleoside triphosphates (rNTPs) (6–8). Its activity is upregulated after a cell enters the S phase, and it is downregulated outside of the S phase to reduce dNTP levels in nonreplicating cells (6, 9). Another family of enzymes known as deoxyguanosine triphosphate triphosphohydrolases (Dgts) regulates intracellular dNTP levels by hydrolyzing dNTPs into deoxynucleosides and triphosphates (5, 10). Dgts are present in all domains of life, but their physiological purpose remains less defined.

Few Dgts have been characterized, and differences in their catalytic mechanisms have led to nebulous conclusions about their functions (11–19). Dgts are members of the HD hydrolase superfamily (20, 21). They harbor an HD motif that coordinates a divalent cation necessary for catalysis. All Dgts hydrolyze dNTPs through the same mechanism, but individual enzymes display a variety of substrate preferences. Some enzymes also require the binding of dNTPs at specific allosteric sites to activate dNTP hydrolysis, but the need for allosteric activation varies among different enzymes and depends on the identity of the cation present in the active site (16, 17, 22).

**Fig 1:**
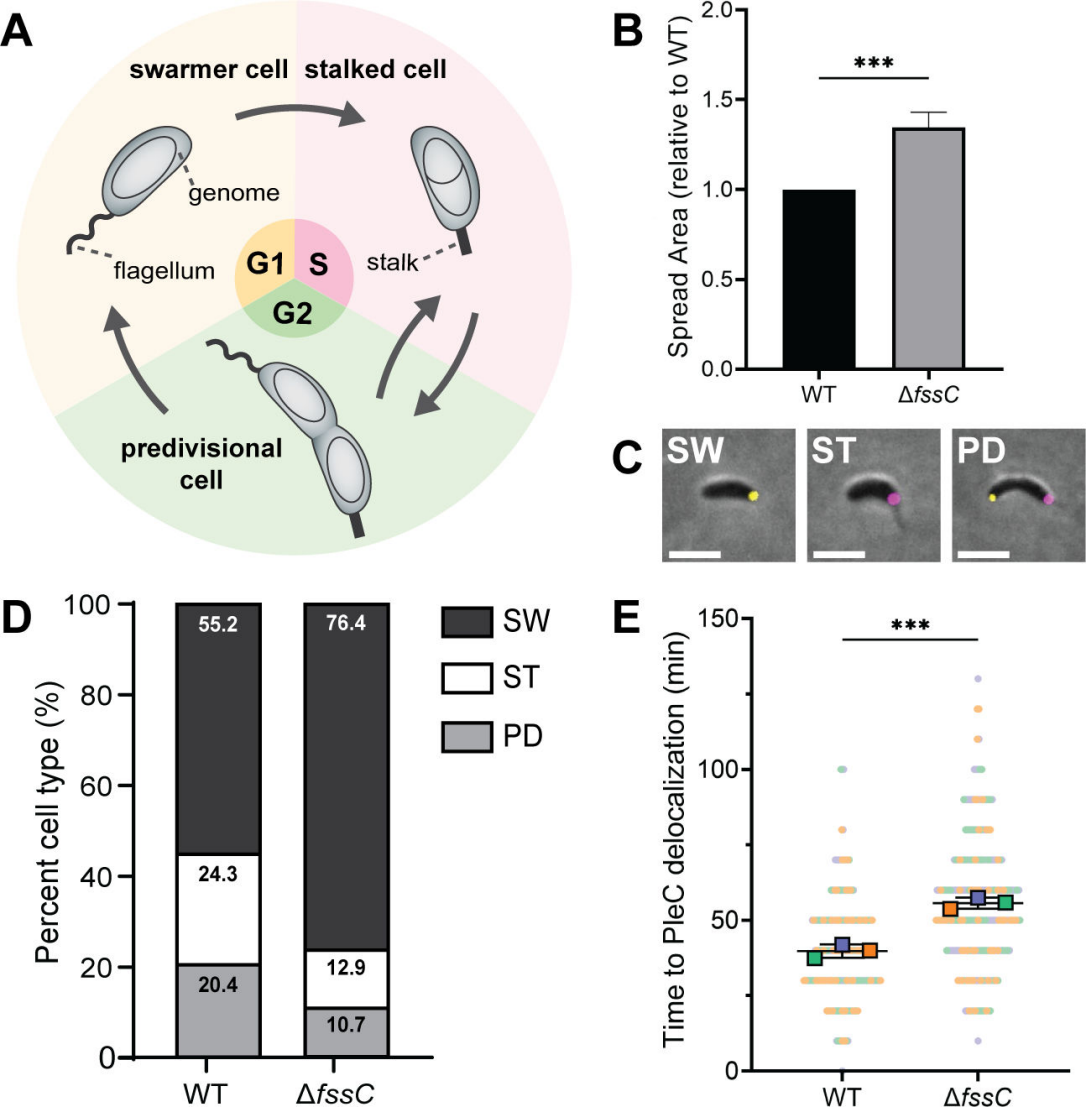
*fssC* promotes the swarmer-stalked transition. (**A**) The dimorphic life cycle of *C. crescentus*. Motile swarmer cells are arrested in the G1 phase of the cell cycle and must differentiate into sessile stalked cells before entering S phase. (**B**) The relative areas of WT and ∆*fssC* in a soft agar assay are shown. The ∆*fssC* mutant spreads 30% more efficiently than WT through semi-solid medium. Error bars represent the standard deviation of the mean for four biological replicates. The genomic locus/GenBank accession number for *fssC* in NA1000 and CB15 strains is CCNA_02087/ACL95552.1 and CC_2008/AAK23983.1, respectively. FssC’s UniProt accession number is Q9A6S5, and its UniProt name is DGTL1_CAUVC. (**C**) Example micrographs showing PleC-Venus (yellow) and DivJ-mKate (magenta) localization throughout the cell cycle. Scale bar is 2 µm. SW, swarmer; ST, stalked; and PD, predivisional. (**D**) Measuring the localization of PleC-Venus and DivJ-mKate with fluorescent microscopy differentiates the phases of the *C. crescentus* life cycle. The ∆*fssC* mutant has a higher percentage (76.4%) of swarmer cells compared to WT (55.2%, *P =* 0.0067). Images were collected from unsynchronized CB15 populations in the early exponential phase. Each bar represents *n* > 1,000 cells collected over three biological replicates. Standard deviations are reported in Table S2. (**E**) ∆*fssC* has, on average, a 15-min delay in the disappearance of its PleC-Venus foci. Superplots show the means for three biological replicates. Each replicate comprises *n* > 55 cells. All statistical comparisons were made using an unpaired *t*-test. ***P < 0.001.

Dgts have proposed roles in DNA repair (23), viral defense (10, 24–28), and eukaryotic cell cycle regulation (10, 12, 29), but a unified role for these enzymes remains elusive. We identified a Dgt from *Caulobacter crescentus* that establishes these enzymes as cell cycle regulators in bacteria. *C. crescentus* is a dimorphic bacterium that serves as an excellent model for studying the cell cycle (Fig. 1A) (30). There are two distinct *C. crescentus* cell types: motile swarmer cells and sessile stalked cells. Swarmer cells are considered replication incompetent; they initiate a developmental program to differentiate into stalked cells before entering the S phase. Division in *C. crescentus* is asymmetric and yields one cell of each type. The stalked cell can immediately reenter the S phase, but the swarmer cell will return to the G1 phase and repeat the cycle (31).

In *C. crescentus,* entry into S phase is controlled by the response regulator CtrA and the initiation factor DnaA (32, 33). CtrA is phosphorylated in swarmer cells and binds to the chromosomal origin of replication (*oriC*) to prevent the binding of DnaA (34). CtrA is deactivated during the swarmer-stalked transition through dephosphorylation and regulated proteolysis, allowing DnaA to bind *oriC* and promote the initiation of DNA replication (35). Timely modulation of CtrA activity allows entry into S phase to be coordinated with the swarmer-stalked morphological transition.

We identified the *C. crescentus* Dgt in a genetic screen designed to identify surface-sensing genes (36). Swarmer cells use their flagellum to physically sense solid surfaces and activate signaling pathways that lead to surface attachment (36–38). Our group identified a panel of genes that are required to activate a surface response when the flagellum is disrupted. These *flagellar-signaling suppressor* (*fss*) genes are predicted to activate surface adhesion downstream of surface sensing. One of the new surface-sensing genes we identified, which we named *fssC*, encodes a putative Dgt (Fig. S1). In this study, we show that *fssC* encodes a Dgt that directly hydrolyzes dNTPs to restrict their intracellular concentrations. Deleting *fssC* delays entry into S phase, as evidenced by a delay in the initiation of DNA replication, and this effect occurs independently of CtrA. We propose that Dgts have an important role in both prokaryotic and eukaryotic cell cycle regulation.

## RESULTS

### *∆fssC* mutants have an elongated swarmer phase

*C. crescentus* cells migrate in semi-solid medium by using flagella to chemotax through the agar matrix. Deleting *fssC* causes cells to spread 30% more efficiently than the WT strain (Fig. 1B). Given that *C. crescentus* is only motile during the swarmer phase of the cell cycle, this hyper-spreading phenotype is suggestive of a delay in the swarmer-stalked transition (39). We developed a fluorescence microscopy-based tool to quantify the proportion of swarmer cells in WT and ∆*fssC* populations. The histidine kinases PleC and DivJ were each fused to different fluorescent tags at their native loci. PleC localizes to the flagellar pole of swarmer cells and was fused to the yellow fluorescent protein Venus. DivJ localizes to the stalked pole of stalked cells and was fused to the red fluorescent protein mKate (40). These reporters allow for the visualization of each stage of the *C. crescentus* life cycle (Fig. 1C). The *pleC-venus* and *divJ-mKate* alleles slightly altered the motility phenotypes of WT and ∆*fssC*, but the ∆*fssC* hyper-spreading phenotype was maintained (Fig. S2). We imaged populations of WT and ∆*fssC* in the *pleC-venus divJ-mkate* background and binned individual cells based on their PleC and DivJ localization. The ∆*fssC* mutant had a significantly higher proportion of swarmer cells (76.4%) compared to WT (55.2%), suggesting that this strain has an elongated swarmer phase (Fig. 1D).

Live cell microscopy was performed on unsynchronized cells to directly measure the duration of the swarmer phase in the ∆*fssC* mutant (Fig. 1E). WT and ∆*fssC* strains harboring *pleC-venus* were immobilized on agarose pads, and individual cells were imaged over a 3-hour time-lapse experiment. The time required for PleC-Venus to delocalize in newly divided cells was recorded. PleC-Venus foci delocalize, on average, 15 min later in ∆*fssC* cells than in the WT background, further suggesting that *fssC* promotes the swarmer-stalked transition.

### FssC hydrolyzes dNTPs *in vitro*

The *fssC* gene encodes a predicted Dgt. A select group of Dgt homologs has been characterized, and individual representatives display a variety of substrate preferences and activation mechanisms. The Dgts from *Escherichia coli* and *Leeuwenhoekiella blandensis* display strict specificity for dGTP and do not require allosteric activation (11, 13). TT1383 from *Thermus thermophilus* and EF1143 from *Enterococcus faecalis* hydrolyze all four canonical dNTPs but require allosteric activation by specific dNTP substrates (14, 16, 17). Analysis of Dgts crystallized with different dNTPs indicates that activating dNTPs bind to allosteric sites to enhance affinity for dNTP substrates at the catalytic site (13, 16, 17). However, TT1383 and EF1143 only require activation when the reaction buffer is supplemented with Mg^2+^ as the divalent cation. Replacing Mg^2+^ with Mn^2+^ circumvents the requirement for activation (16, 22).

**Fig 2:**
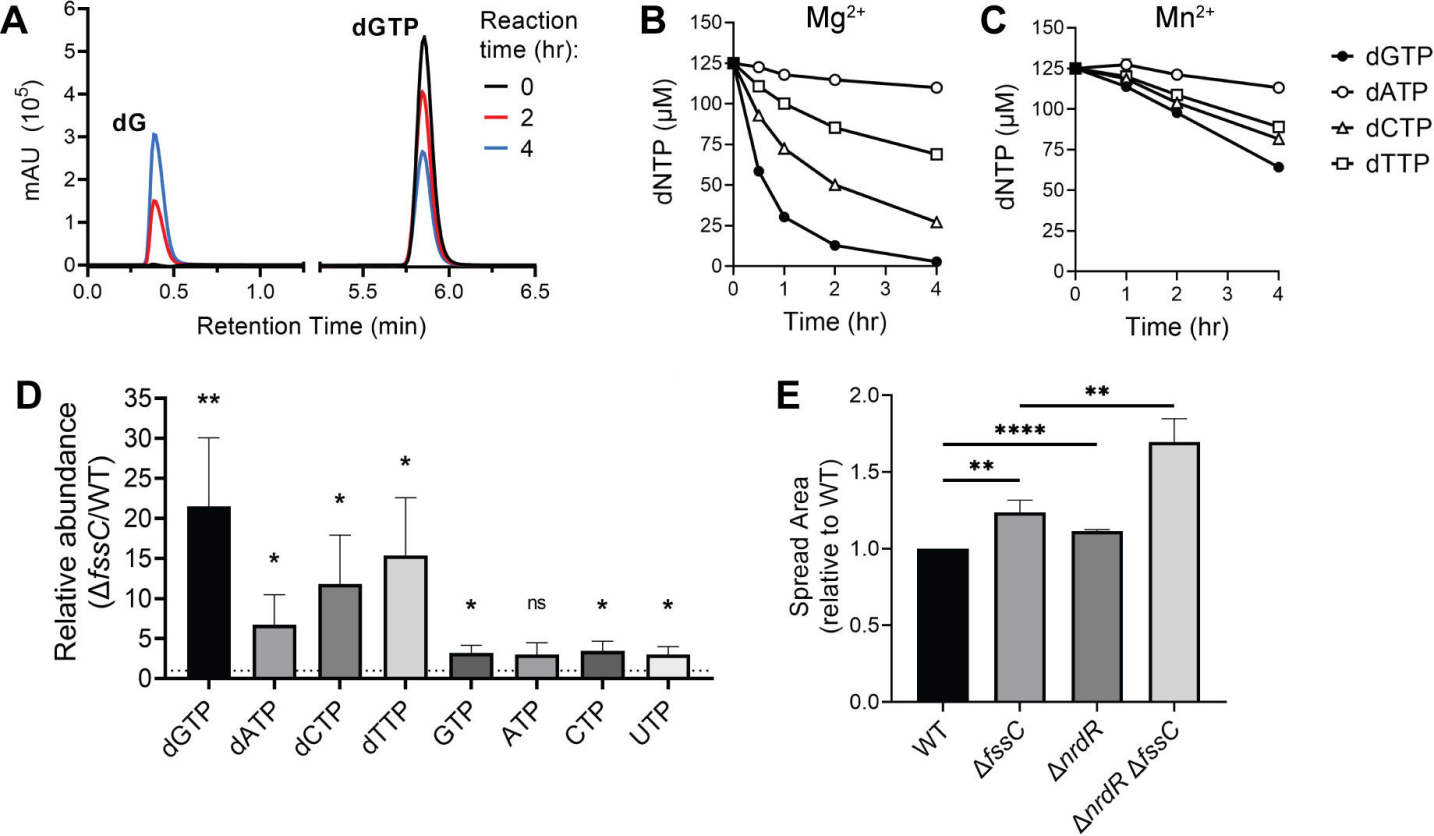
*fssC* encodes a deoxyguanosine triphosphate triphosphohydrolase (Dgt). (**A**) dNTP hydrolysis was analyzed by anion exchange chromatography. Purified FssC was incubated with dGTP for 0 (black), 2 (red), and 4 hours (blue) in reaction buffer supplemented with Mn^2+^. The dGTP substrate elutes at 5.85 min, and the dG product elutes at 0.388 min. (**B and C**) FssC hydrolyzes all four canonical dNTPs *in vitro* with a preference for dGTP (dGTP > dCTP > dTTP > dATP). FssC was incubated with the four dNTPs mixed together (125 µM each, 500 µM total) in buffer containing Mg^2+^ (**B**) or Mn^2+^ (**C**) as the divalent cation. Error bars represent the standard deviation of the mean for three replicates. (**D**) The ∆*fssC* mutant has higher intracellular dNTP levels than WT. Nucleotides were extracted from cell cultures and quantified by LC/MS. dGTP levels are, on average, 20 times higher in ∆*fssC* compared to WT. dATP, dCTP, and dTTP are also elevated but to a lesser extent. rNTP abundance is only slightly increased (~3× higher in ∆*fssC*). Error bars represent the standard deviation of the mean for three biological replicates. (**E**) The relative areas of WT, ∆*fssC*, ∆*nrdR*, and ∆*fssC* ∆*nrdR* in a soft agar assay. The increased spreading of the ∆*nrdR* mutants further supports the model that elevated dNTPs are the cause of the ∆*fssC* mutant’s elongated swarmer phase. Error bars represent the standard deviation of the mean for three biological replicates. All statistical comparisons were made using an unpaired *t*-test. *P < 0.05, **P < 0.01, and ****P < 0.0001.

We purified recombinant FssC and used anion exchange chromatography to measure its ability to hydrolyze various nucleotide substrates (Fig. 2A). FssC hydrolyzed each of the four dNTPs (dGTP, dATP, dCTP, and dTTP), as measured by a decrease in the concentration of the dNTP substrate. The products of the reactions were identified as deoxynucleosides, confirming FssC is a triphosphohydrolase (Fig. S3). Activity assays were performed with both individual dNTPs (Fig. S4) and with combinations of dNTPs. Two different reaction buffers were used (Fig. 2B and C). The first buffer contained Mg^2+^ as the divalent cation, and the second contained Mn^2+^. FssC demonstrates a kinetic preference for dGTP in both conditions. However, dNTP hydrolysis only occurs in the Mg^2+^ buffer when FssC is incubated with dATP and at least one other dNTP (Table S1; Fig. S4), suggesting that FssC requires activation by dATP when Mg^2+^ serves as the catalytic metal ion.

We tested FssC’s activity with a panel of potential nucleotide substrates. Assays were performed in three conditions: buffer supplemented with Mn^2+^, with Mg^2+^, or in activating conditions with Mg^2+^ and dATP. We examined deoxynucleotides (dGDP and dGMP), ribonucleotides (GTP), and the signaling nucleotides c-di-GMP, pppGpp, ppGpp, and pGpp. Hydrolysis by FssC was not detected for any of these substrates (Fig. S5).

We constructed a catalytically inactive FssC mutant by mutating the HD motif that coordinates the active site cation. The FssC H102A D103A variant was unable to hydrolyze dNTPs (Fig. S6A). We used the H102A D103A variant to test if FssC’s hydrolysis activity was required for the enzyme to stimulate cell cycle progression. Expressing *fssC* from its native promoter at an ectopic locus in the ∆*fssC* mutant restores the wild-type motility phenotype in soft agar, but the hyper-spreading phenotype persists when the inactive H102A D103A variant is expressed (Fig. S6B). This demonstrates that FssC’s catalytic activity is necessary for regulating the swarmer-stalked transition. Indeed, ectopically expressing the H102A D103A mutant does not decrease the percentage of swarmers in the ∆*fssC* strain, while expressing wild-type *fssC* does (Fig. S6C). We conclude that dNTP hydrolysis by FssC is required for *C. crescentus* to efficiently progress through the cell cycle.

### FssC restricts intracellular dNTP concentrations

We used targeted metabolomics to examine the role of *fssC* in maintaining intracellular dNTP concentrations. Nucleotides were extracted from WT and ∆*fssC* cultures and analyzed by LC/MS to determine their relative abundances. The ∆*fssC* mutant has higher dNTP levels than WT (Fig. 2D), and the relative abundances mirror the *in vitro* substrate preference of the FssC enzyme (dGTP > dCTP > dTTP > dATP). The levels of rNTPs were also two to three times higher in ∆*fssC*. While it is possible that the FssC enzyme is more promiscuous *in vivo* than the *in vitro* hydrolysis assays indicate, we favor the explanation that the elevated dNTPs could alter the flux of nucleotide metabolism and lead to a slight increase in rNTPs that is not a direct result of FssC activity.

We also increased dNTP levels through an *fssC*-independent mechanism. RNR is transcriptionally regulated by the repressor NrdR (6). Deleting *nrdR* should, therefore, increase RNR expression and dNTP synthesis. If elevated dNTPs in the ∆*fssC* strain elongate the swarmer phase, then a ∆*nrdR* mutant should also exhibit a hyper-spreading phenotype on soft agar. As expected, the ∆*nrdR* strain spreads 10% more efficiently than WT, and a ∆*nrdR* ∆*fssC* double mutant showed a synergistic hyper-spreading phenotype (Fig. 2E). These results demonstrate that FssC promotes the swarmer-stalked transition by restricting intracellular dNTP concentrations.

### FssC does not affect the elongation phase of DNA replication

**Fig 3:**
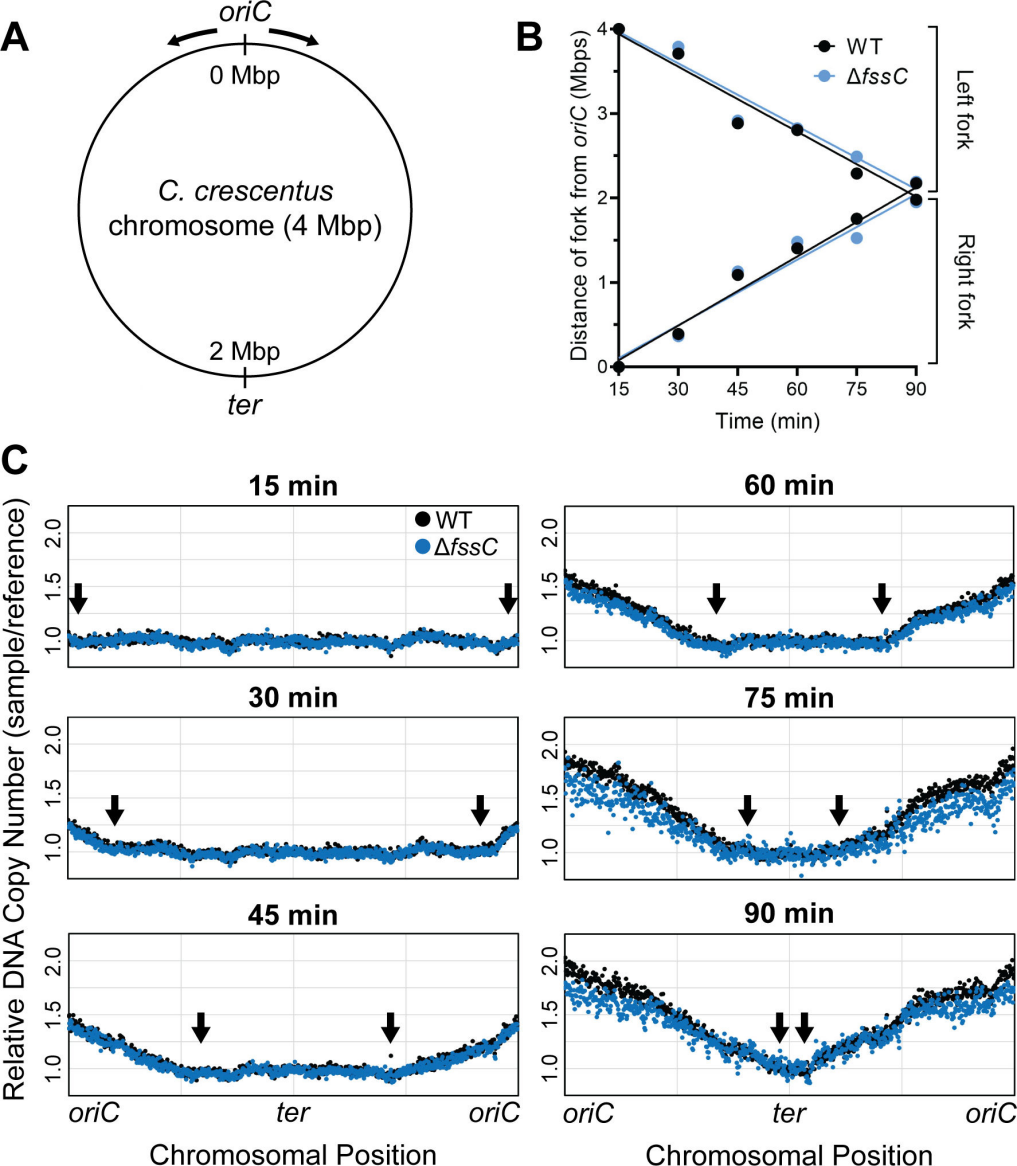
The ∆*fssC* mutant has a wild-type rate of DNA replication. (**A**) The circular chromosome of *C. crescentus* is 4 Mbp in length. The origin of replication (*oriC*), the terminus (*ter*) region, and the direction of the replication forks (black arrows) are shown. (**B**) Positions of the left and right replication forks are plotted as a function of time for WT (black) and ∆*fssC* (blue). Replisomes at the left and right forks in WT synthesize DNA at a rate of 428 ± 51 and 455 ± 39 bp/s, respectively. The left and right forks in ∆*fssC* move at a rate of 414 ± 50 and 432 ± 54 bp/s, respectively. Line of best fit is shown for each fork. Slopes are not significantly different (*P* = 0.8547 for right forks and *P* = 0.7338 for left forks). *P*-values were determined by an extra sum-of-squares *F*-test. (**C**) Replication was monitored in synchronized NA1000 cells with a high-throughput sequencing approach. Read counts for each chromosomal position were normalized to *t* = 0 to calculate relative copy number across the chromosome. Replication forks (black arrows) are at the interface between replicated and unreplicated DNA (43). Data are representative of two replicates.

Elevated or imbalanced dNTP levels can influence the rate of DNA replication (1–4, 41). High-throughput sequencing was used to measure the DNA replication rate in WT and ∆*fssC* cells (42) of a synchronizable *C. crescentus* strain (NA1000). Synchronized swarmer cells were isolated from a density gradient, and genomic DNA was sequenced at various time points after the cells were reintroduced into growth medium. The relative read coverage was plotted as a function of chromosome position to identify the locations of the replication forks (Fig. 3).

Replisomes on the left and right forks of the *C. crescentus* chromosome synthesize DNA at rates comparable to those in *Escherichia coli* and *Bacillus subtilis* (43, 44). The rate of replication in the ∆*fssC* mutant is indistinguishable from the WT background. This experiment was also performed with cells grown in M2X media (Fig. S7). We reasoned that cells in minimal media would grow slower and that any replication differences between the two strains would be exacerbated. The results were comparable to the cells grown in PYE, confirming that the replication rates of WT and ∆*fssC* are identical. We conclude that elevated dNTP levels delay the swarmer-stalked transition through a mechanism independent of replication elongation.

### Segregation of the origin of replication (*oriC*) is delayed in *∆fssC*

**Fig 4:**
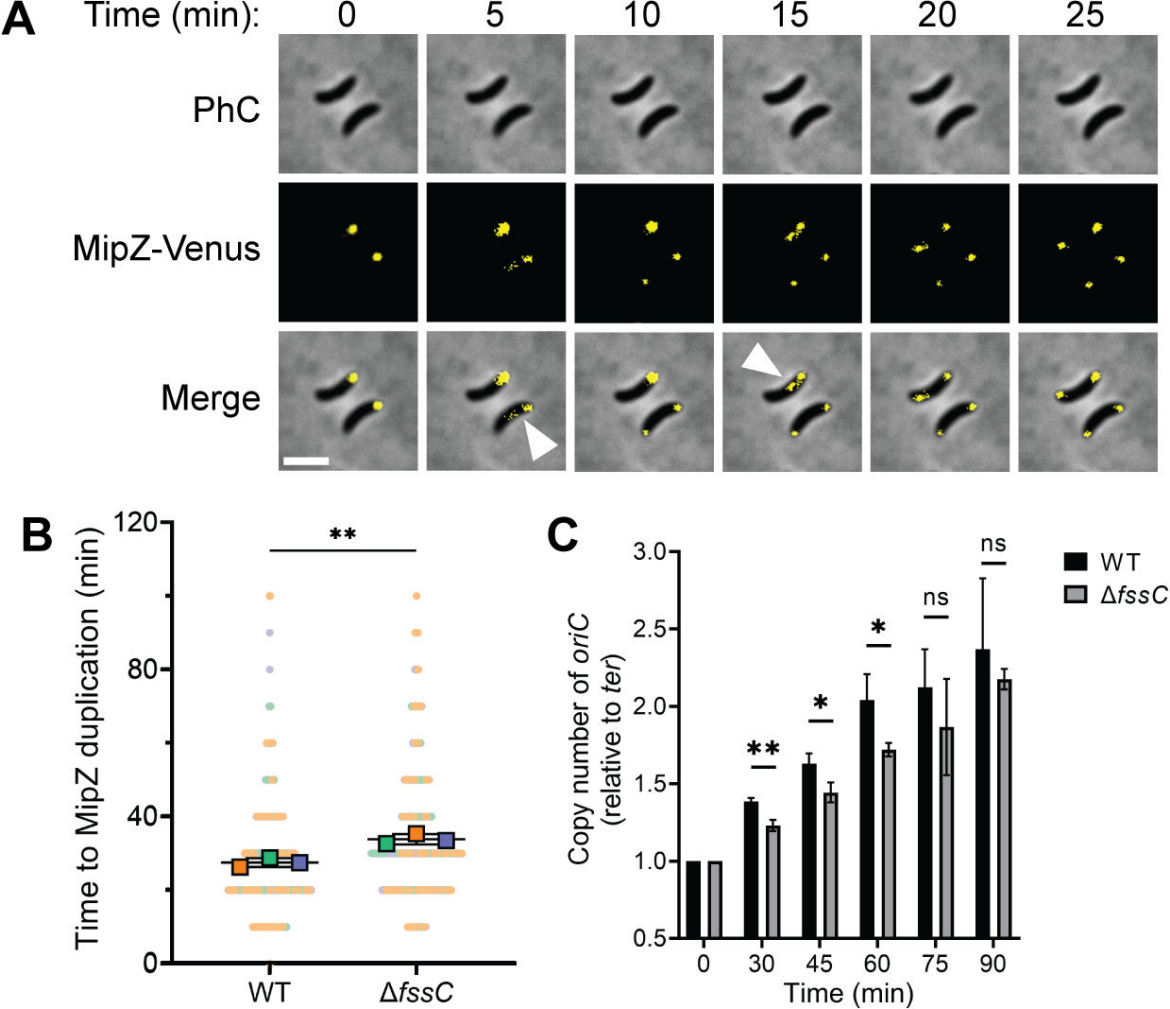
*fssC* promotes timely segregation and duplication of *oriC*. (**A**) Representative micrographs showing the duplication of Venus-MipZ foci. Scale bar is 2 µm. (**B**) ∆*fssC* has, on average, a 6-min delay in MipZ duplication compared to WT. Superplots show the means for three biological replicates. Each replicate comprises *n* > 60 cells. (**C**) Relative copy number of *oriC* in synchronized populations over time was determined by quantitative PCR. Primers were designed for *oriC* and the *ter* region (see Fig. 3A). The amount of *oriC* in each sample was normalized to the amount of *ter* and then to *t* = 0. Error bars represent the standard deviation of the mean for three biological replicates. Unpaired *t*-tests were used to determine statistical significance. *P < 0.05 and **P < 0.01.

We next tested if the *∆fssC* mutant has a delay in chromosome segregation. MipZ is a protein that associates with the centromere region near *oriC* on the *C. crescentus* chromosome (45). Fusing MipZ to a fluorescent tag allows partitioning of the origin region to be tracked in live cells (Fig. 4A). We recorded the time required for newly divided cells to duplicate their Venus-MipZ foci as a measure of when the chromosomes begin to segregate. On average, ∆*fssC* cells duplicated their Venus-MipZ foci 6 min later than WT cells (Fig. 4B). A similar experiment was performed with NA1000 cells synchronized in the swarmer phase (Fig. S8). Venus-MipZ duplicates, on average, 5 min later in synchronized ∆*fssC*. These results indicate that the ∆*fssC* mutant has a delay in segregation of the origin region despite having a replication rate identical to WT.

### Initiation of DNA replication is delayed in *∆fssC*

We predicted that the delay in the segregation of *oriC* reflected a delay in the initiation of DNA replication. A closer look at the high-throughput sequencing data (Fig. 3C; Fig. S7B) supports this hypothesis. The copy number of *oriC* in the ∆*fssC* mutant is below that of WT at all time points.

We directly investigated the timing of replication initiation by measuring the relative copy number of *oriC* via quantitative PCR (qPCR). WT and ∆*fssC* cells were synchronized in the swarmer phase, and qPCR was performed on the *oriC* and the *ter* regions of the chromosome (Fig. 3A) to measure the *oriC*/*ter* ratio over time. When grown in PYE, the ∆*fssC* mutant has less *oriC* present than WT for up to 75 min, at which point both strains have fully duplicated their origins and reached a copy number of 2N (Fig. 4C). qPCR was also performed on samples grown in M2X, yielding similar results (Fig. S7C). These data support the model that ∆*fssC* has a delay in the initiation of DNA replication and suggest that dNTP hydrolysis by FssC plays an important role in regulating entry into S phase.

### The effect of *fssC* is independent of other cell cycle regulators, including CtrA

**Fig 5:**
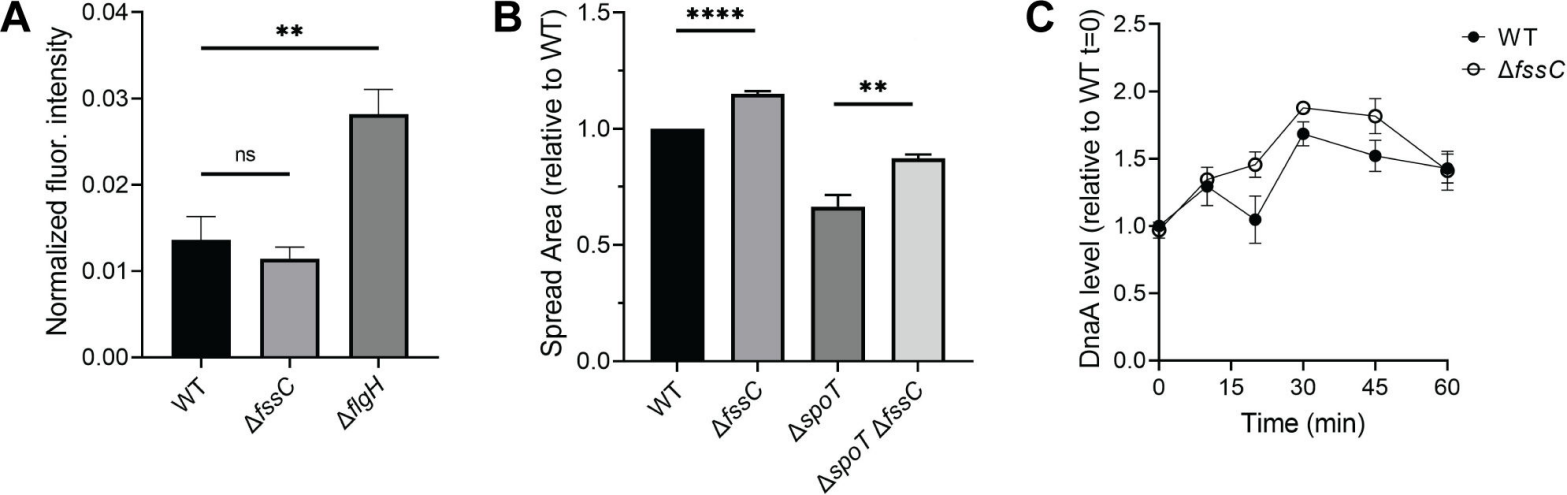
The effect of *fssC* on canonical *C. crescentus* cell cycle regulators. (**A**) WT and ∆*fssC* strains have similar levels of c-di-GMP. c-di-GMP levels were measured using the fluorescent biosensor cdGreen2 (47). An ∆*flgH* mutant (see Fig. S1) is shown as a positive control. (**B**) Deleting *fssC* in the ∆*spoT* background causes a hyper-spreading phenotype on soft agar compared to the ∆*spoT* single deletion. (**C**) DnaA protein levels throughout the cell cycle, starting with synchronized swarmer cells at *t* = 0. DnaA levels are unchanged in the ∆*fssC* mutant. The corresponding immunoblots can be found in the supplemental material (Fig. S9). All error bars represent the standard deviation of the mean for three biological replicates. All statistical comparisons were made using an unpaired *t*-test. **P < 0.01 and ****P < 0.0001.

We next investigated if the *fssC-*signaling pathway intersects with established pathways for *C. crescentus* cell cycle regulation. The second messengers c-di-GMP and ppGpp are known to regulate the G1-S phase transition (39, 46). FssC did not hydrolyze these nucleotides *in vitro* (Fig. S5), but we sought to confirm that they do not affect the ∆*fssC* cell cycle phenotype. c-di-GMP levels increase during the swarmer-stalked transition to promote cellular differentiation and the initiation of DNA replication (46). We measured c-di-GMP levels in WT and ∆*fssC* backgrounds using the protein biosensor cdGreen2 (47). The fluorescence intensity of cdGreen2 in ∆*fssC* was indistinguishable from WT (Fig. 5A), indicating that global c-di-GMP levels do not change in the absence of *fssC*.

**Fig 6:**
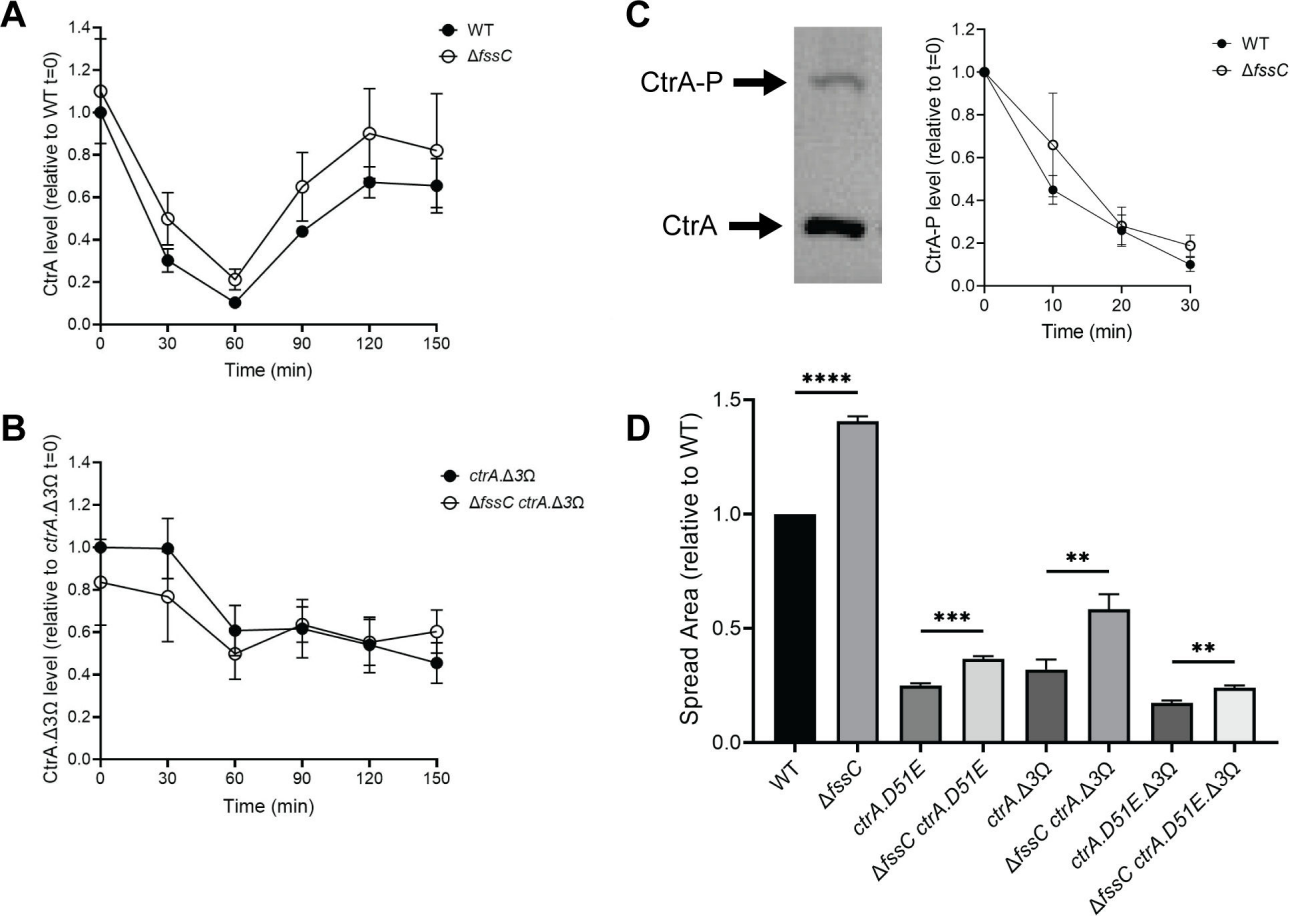
The FssC signaling pathway is independent of CtrA. (**A**) CtrA protein levels throughout the cell cycle, starting with synchronized swarmer cells at *t* = 0. The ∆*fssC* mutant has moderately higher CtrA levels than WT after 30 min. (**B**) The stabilized CtrA.∆3Ω variant is present in WT and ∆*fssC* backgrounds at equal levels throughout the cell cycle. (**C**) Phos-Tag gel electrophoresis separates phosphorylated and unphosphorylated CtrA. WT and ∆*fssC* have identical rates of CtrA dephosphorylation. All immunoblots can be found in the supplemental material (Fig. S10, S11, and S14). (**D**) Deleting *fssC* in *ctrA* mutants containing a phosphomimetic (*ctrA.D51E*) or stabilized (*ctrA.∆3*Ω) *ctrA* allele causes a hyper-spreading phenotype on soft agar. A representative soft agar image can be found in the supplemental material (Fig. S15). All error bars represent the standard deviation of the mean for three biological replicates. All statistical comparisons were made using an unpaired *t*-test. **P < 0.01, ***P < 0.001, and ****P < 0.0001.

ppGpp delays the G1-S phase transition in *C. crescentus* (39). We constructed a ∆*spoT* mutant that is unable to synthesize ppGpp (48). Deleting *spoT* decreases spreading on soft agar in the WT background, but a ∆*spoT* ∆*fssC* double mutant still exhibits hyper-spreading compared to the single ∆*spoT* mutant (Fig. 5B). This result indicates that the ∆*fssC* cell cycle phenotype is independent of ppGpp.

We used immunoblotting to examine how *fssC* affects DnaA and CtrA, two main regulators of replication initiation in *C. crescentus* (Fig. 5C and 6A; Fig. S9 and S10) (32–35). DnaA levels remain relatively constant during the cell cycle (49, 50), and deleting *fssC* has minimal effects on the concentration of DnaA (Fig. 5C). CtrA is degraded with similar timing in WT and ∆*fssC* cells, but the ∆*fssC* mutant has a modest increase in CtrA levels during the swarmer-stalked transition (Fig. 6A; Fig. S9). Moderately higher CtrA levels are not predicted to affect the cell cycle because both the proteolysis and dephosphorylation of CtrA must be disrupted to delay S phase (35, 51, 52). We used Phos-Tag gel electrophoresis (53, 54) to show that deleting *fssC* does not cause a delay in CtrA dephosphorylation (Fig. 6C; Fig. S11). Additionally, cell shape analysis determined that ∆*fssC* cells do not exhibit the filamentation phenotype seen in strains with stabilized CtrA (Fig. S12) (35). These results indicate that *fssC* has minimal effects on DnaA and CtrA levels and suggest that *fssC* promotes the initiation of DNA replication through a separate mechanism.

To confirm that the elongated swarmer phase observed in the ∆*fssC* mutant is independent of *ctrA*, we replaced *ctrA* with phosphomimetic (*ctrA.D51E*) and stabilized (*ctrA.*∆*3*Ω) *ctrA* alleles in WT and ∆*fssC* backgrounds (Fig. 6B; Fig. S13 and S14) (35). The *ctrA* mutants grow slower than WT cells in soft agar, but deleting *fssC* in any of the *ctrA* mutant backgrounds still causes hyper-spreading (Fig. 6D; Fig. S15). This result demonstrates that the ∆*fssC* cell cycle phenotype is not dependent on the phosphorylation or the proteolysis of CtrA. We conclude that CtrA is not a direct target of *fssC*-dependent cell cycle regulation and that elongation of the swarmer phase occurs independently of CtrA in the ∆*fssC* mutant.

## DISCUSSION

dNTP levels are precisely regulated throughout the cell cycle to avoid DNA damage and promote efficient DNA replication (1–5). Over the last decade, it has become clear that Dgts play an important role in regulating dNTP levels (5, 10). These enzymes reduce dNTP concentrations by hydrolyzing dNTPs into deoxynucleosides, but their physiological purpose remains debated.

We have characterized a Dgt from *C. crescentus* called *flagellar signaling suppressor C* that regulates the length of the G1 phase of the cell cycle. *In vitro* characterization confirmed that the FssC enzyme shows dNTP triphosphohydrolase activity with a kinetic preference for hydrolyzing dGTP (Fig. 2). Hydrolysis assays were performed with an excess of dNTP substrates, and we can infer turnover numbers for FssC. We suspect that assays performed with a mixture of the four dNTPs and Mg^2+^ as the divalent cation most closely resemble physiological FssC conditions. The *k*_cat_ of FssC for dGTP under these conditions is 0.26 ± 0.023 s^−1^. This value is comparable to that of other characterized Dgts, including the human Dgt homolog SAMHD1 that has an analogous cell cycle phenotype (13–15, 55, 56). The dNTP hydrolysis activity of FssC is also relevant *in vivo*. We confirmed that dNTP levels are elevated in the ∆*fssC* mutant using targeted metabolomics (Fig. 2D). All four canonical dNTPs are at least five times higher in ∆*fssC*, and dGTP was the most elevated, with levels 20 times higher than WT.

We suspected that *fssC* may have a role in controlling the cell cycle after examining motility in semi-solid agar (Fig. 1B). The ∆*fssC* mutant migrates 30% more efficiently than WT, and we predicted that this result was caused by an elongated swarmer phase. Indeed, fluorescence microscopy experiments showed a delay in the delocalization of PleC-Venus when *fssC* is deleted (Fig. 1C through E). A catalytically inactive *fssC* allele (H102A D103A) is unable to restore the WT phenotype in soft agar or the length of the swarmer phase, showing that dNTP hydrolysis by FssC is required for proper cell cycle progression (Fig. S6). Increasing dNTP levels through a mechanism independent of *fssC* also resulted in a hyper-spreading phenotype on soft agar, further demonstrating that elevated dNTP pools delay entry into the stalked phase (Fig. 2E).

The ∆*fssC* mutant also shows a delay in the G1-S phase transition. Time-lapse microscopy of WT and ∆*fssC* strains harboring a fluorescent Venus-MipZ fusion showed that the ∆*fssC* mutant has a delay in the segregation of *oriC* (Fig. 4B; Fig. S8). This led us to hypothesize that the deletion of *fssC* causes a delay in the initiation of DNA replication. We determined the relative copy number of *oriC* by performing qPCR on genomic DNA and found that the ∆*fssC* mutant had less *oriC* than WT (Fig. 4C; Fig. S7C). This result indicates that ∆*fssC* cells, on average, duplicate their *oriC* later than WT cells. It is possible that *fssC* does not directly act on the initiation of DNA replication and that the observed delay into S phase is a product of delayed stalked cell differentiation. However, our data suggest that completing stalked cell morphogenesis is not a strict prerequisite for DNA replication. Our time-lapse microscopy experiments demonstrate that PleC delocalization occurs after MipZ duplication in WT cells (40 and 27 min on average, respectively). We favor a model in which elevated dNTP levels in the ∆*fssC* mutant directly delay the initiation of DNA replication.

An analogous phenotype has previously been associated with elevated dNTP concentrations in human fibroblasts, *Trypanosoma brucei*, and budding yeast (10, 12, 29). These findings are counterintuitive from a biochemical perspective. As substrates for DNA polymerase, elevated dNTPs might be expected to promote DNA replication. The opposite has now been observed in two different domains of life. We propose that elevated dNTPs target the initiation step of DNA replication and that Dgts reduce dNTP levels for efficient progression of the cell cycle.

The mechanism by which elevated dNTPs delay the initiation of DNA replication remains undefined. Some evidence suggests that high dNTPs delay the activation of the replicative helicase in yeast (29). The exact sequence of *fssC*-dependent cell cycle regulation is unknown, but our analysis indicates that *fssC* promotes replication initiation downstream of CtrA and DnaA (Fig. 5 and 6). We propose that elevated dNTPs might target the helicase DnaB or the helicase loader DciA. It is also unclear how delaying replication initiation could delay the swarmer-stalked transition, but this phenotype has been observed previously in *C. crescentus* (57). We suspect there is a regulatory mechanism connecting the initiation of DNA replication to stalked cell differentiation that is presently still undefined.

Lowering dNTP concentrations can also affect the replication of invading viral genomes. Some studies have proposed that Dgts hydrolyze dNTPs primarily as an antiviral strategy (24, 25). The depletion of dNTP pools can limit viral replication, and some bacterial Dgts are encoded next to other known phage defense genes (24). However, not all Dgts improve a host’s resistance to viral infection (24). We found that *fssC* does not influence the susceptibility of *C. crescentus* to ɸCbK infection (Fig. S16). We propose that increased viral defense is an indirect effect of dNTP hydrolysis and that the primary role of Dgts is to promote entry into S phase.

We have shown that *fssC* promotes cell cycle progression in *C. crescentus.* Elevated dNTP levels delay the swarmer-stalked transition, and we propose that *fssC* promotes the initiation of DNA replication through dNTP hydrolysis. Analogous phenotypes have been observed in eukaryotic organisms, and we predict that Dgt-dependent cell cycle regulation is widespread across the tree of life.

## MATERIALS AND METHODS

### Bacterial strains, growth, and genetic manipulation

The strains used in this study are listed in Table 1. Plasmids (Table 2) were developed with PCR, restriction digestion, and Gibson assembly unless otherwise stated. Primer sequences are available upon request. The genomic locus/GenBank accession numbers for *fssC* in NA1000 and CB15 strains are CCNA_02087/ACL95552.1 and CC_2008/AAK23983.1, respectively. FssC’s UniProt accession number is Q9A6S5, and its UniProt name is DGTL1_CAUVC. *E. coli* was grown in LB medium at 37°C and supplemented with 50 µg/mL kanamycin when necessary. *C. crescentus* was grown in PYE medium or M2 minimal media supplemented with 0.15% xylose (M2X) at 30°C. All experiments were performed in PYE unless otherwise stated. Liquid and solid PYE media were supplemented with 5 and 25 µg/mL kanamycin, respectively, when required. Plasmids were transformed into *C. crescentus* by electroporation. Gene deletions and insertions were constructed with a two-step approach using *sacB-*based counterselection.

**TABLE 1:** Plasmids used in this study

Plasmid	Description	Antibiotic	Reference
pNPTS138	Suicide plasmid for making unmarked deletions in *C. crescentus;* carries *sacB* for counterselection.	Km	M. R. Alley, unpublished
pDH118	pMT585, pMTLS4259, integrating vector for xylose inducible expression of C-terminally GFP-tagged proteins in *Caulobacter.*	Km	(58)
pDH804	To delete *fssC*; Gibson cloning of fused upstream and downstream regions of CC_2008.	Km	This work
pDH1161	To fuse fluorescent mVenus protein to C-terminus of PleC; Gibson cloning of *mvenus* fused between 3′ end and downstream region of CC_2482.	Km	This work
pDH1209	To fuse fluorescent mKate2 protein to C-terminus of DivJ; Gibson cloning of *mkate2* fused between 3′ end and downstream region of CC_ 1063.	Km	This work
pDH430	Modified pET28a plasmid that includes an 8xHis-SUMO tag upstream of multicloning site	Km	This work
pDH815	To overexpress FssC in *E. coli*; 8xHis-SUMO-FssC cloned into pDH430.	Km	This work
pDH1230	To overexpress FssC H102A D103A in *E. coli*; QuikChange mutagenesis of pDH815.	Km	This work
pDH1335	To fuse fluorescent mVenus protein to the N-terminus of MipZ; Gibson cloning of *mvenus* fused between the upstream region and 5′ end of CC_2165.	Km	This work
pDH1158	To integrate *PfssC*-empty at *xyl* locus; Gibson cloning of CC_2008 promoter (88 nt upstream).	Km	This work
pDH1167	To integrate *PfssC-fssC* at *xyl* locus; Gibson cloning of CC_2008 fused to CC-2008 promoter (88 nt upstream).	Km	This work
pDH1224	To integrate *PfssC-fssC* H102A D103A at *xyl* locus; QuikChange mutagenesis of pDH1167.	Km	This work
pDH418	To delete *flgH*; Gibson cloning of fused upstream and downstream regions of CC_2066.	Km	(59)
pDH805	To delete *fliF;* Gibson cloning of fused upstream and downstream regions of CC_0905.	Km	(36)
pDH1396	To delete *nrdR*; Gibson cloning of fused upstream and downstream regions of CC_1358.	Km	This work
pDH1398	To delete *spoT*; fused upstream and downstream regions of CC_1553 synthesized by Azenta.	Km	This work
pDH1397	To integrate cdGreen2.1 at the *xyl* locus; fused to *lacUV5* promoter and *rrnB* terminator. Synthesized by Azenta.	Km	This work
pDH1409	To replace *ctrA* with *ctrA.D51E*; Gibson cloning of *ctrA* ±500 bp followed by mutagenesis (C153G).	Km	This work
pDH1410	To replace *ctrA* with *ctrA.*∆*3*Ω; Gibson cloning of *ctrA* ±500 bp followed by mutagenesis (last three amino acids replaced with Ω-tag).	Km	This work
pDH1411	To replace *ctrA* with *ctrA.D51E.*∆*3*Ω; mutagenesis of pDH1409 (last three amino acids replaced with Ω-tag).	Km	This work

**TABLE 2:** Strains used in this study

Strain	Organism	Genotype	Description	Source
DH103	*C. crescentus* CB15	CB15	Wild type	ATCC 19089
DH1077	*C. crescentus* NA1000	NA1000	Wild type	Poindexter
DH817	*C. crescentus* CB15	∆*fssC*	In-frame deletion of CC_2008	This work
DH1338	*C. crescentus* NA1000	∆*fssC*	In-frame deletion of CC_2008	This work
DH1210	*C. crescentus* CB15	*pleC*::*pleC-venus divJ*::*divJ-mkate2*	Replacement of *divJ* with *divJ-mkate2* and *pleC* with *pleC-venus* in DH103 background	This work
DH1219	*C. crescentus* CB15	∆*fssC pleC*::*pleC-venus divJ*::*divJ-mkate2*	In-frame deletion of CC_2008 in DH1210 background	This work
DH1336	*C. crescentus* NA1000	*mipZ*::*venus-mipZ*	Replacement of *mipZ* with *venus-mipZ* in DH1077 background	This work
DH1337	*C. crescentus* NA1000	∆*fssC mipZ*::*venus-mipZ*	In-frame deletion of CC_2008 in CH49 background	This work
DH1339	*C. crescentus* NA1000	∆*fssC xyl*::*PfssC-*empty *pleC*::*pleC-venus divJ*::*divJ-mkate2*	Integration of pDH1158 into DH1219 background	This work
DH1340	*C. crescentus* NA1000	∆*fssC xyl*::*PfssC-fssC pleC*::*pleC-venus divJ*::*divJ-mkate2*	Integration of pDH1167 into DH1219 background	This work
DH1341	*C. crescentus* NA1000	∆*fssC xyl*::*PfssC-fssC* H102A D103A *pleC*::*pleC-venus divJ*::*divJ-mkate2*	Integration of pDH1224 into DH1219 background	This work
DH1200	*C. crescentus* NA1000	∆*fssC xyl*::*PfssC-*empty	Integration of pDH1158 into DH817 background	This work
DH1201	*C. crescentus* NA1000	∆*fssC xyl*::*PfssC-fssC*	Integration of pDH1167 into DH817 background	This work
DH1226	*C. crescentus* NA1000	∆*fssC xyl*::*PfssC-fssC* H102A D103A	Integration of pDH1224 into DH817 background	This work
DH553	*C. crescentus* CB15	∆*flgH*	In-frame deletion of CC_2066	(59)
DH818	*C. crescentus* CB15	∆*flgH* ∆*fssC*	In-frame deletion of CC_2066 and CC_2008	This work
DH816	*C. crescentus* CB15	∆*fliF*	In-frame deletion of CC_0905	(36)
DH1154	*C. crescentus* CB15	∆*fliF* ∆*fssC*	In-frame deletion of CC_0905 and CC_2008	This work
DH1399	*C. crescentus* NA1000	∆*nrdR*	In-frame deletion of CC_1358	This work
DH1400	*C. crescentus* NA1000	∆*nrdR* ∆*fssC*	In-frame deletion of CC_1358 and CC_2008	This work
DH1401	*C. crescentus* CB15	∆*spoT*	In-frame deletion of CC_1553	
DH1402	*C. crescentus* CB15	∆*spoT* ∆*fssC*	In-frame deletion of CC_1553 and CC_2008	This work
DH1403	*C. crescentus* CB15	*xyl*::*lacUV5-cdGreen2.1-rrnBterm*	Integration of pDH1397 into DH103 background	This work
DH1404	*C. crescentus* CB15	*xyl*::*lacUV5-cdGreen2.1-rrnBterm* ∆*fssC*	Integration of pDH1397 into DH817 background	This work
DH1405	*C. crescentus* CB15	*xyl*::*lacUV5-cdGreen2.1-rrnBterm* ∆*flgH*	Integration of pDH1397 into DH553 background	This work
DH1345	*E. coli* DH5α λpir	DH5α λpir	For Gibson cloning and transformations	
DH1346	*E. coli* C43	C43	For overexpression	Novagen
DH1412	*C. crescentus* CB15	*ctrA*::*ctrA.D51E*	Replacement of *ctrA* with *ctrA.D51E* in DH103 background.	This work
DH1413	*C. crescentus* CB15	*ctrA*::*ctrA*.∆*3*Ω	Replacement of *ctrA* with *ctrA*.∆*3*Ω in DH103 background	This work
DH1414	*C. crescentus* CB15	*ctrA*::*ctrA.D51E*.∆*3*Ω	Replacement of *ctrA* with *ctrA.D51E*.∆*3*Ω in DH103 background	This work
DH1415	*C. crescentus* CB15	∆*fssC ctrA*::*ctrA.D51E*	Replacement of *ctrA* with *ctrA.D51E* in DH817 background	This work
DH1416	*C. crescentus* CB15	∆*fssC ctrA*::*ctrA*.∆*3*Ω	Replacement of *ctrA* with *ctrA*.∆*3*Ω in DH817 background	This work
DH1417	*C. crescentus* CB15	∆*fssC ctrA*::*ctrA.D51E*.∆*3*Ω	Replacement of *ctrA* with *ctrA.D51E*.∆*3*Ω in DH817 background	This work
DH1418	*C. crescentus* NA1000	*ctrA*::*ctrA*.∆*3*Ω	Replacement of *ctrA* with *ctrA*.∆*3*Ω in DH1077 background	This work
DH1419	*C. crescentus* NA1000	∆*fssC ctrA*::*ctrA*.∆*3*Ω	Replacement of *ctrA* with *ctrA*.∆*3*Ω in DH1338 background	This work

### Soft agar motility assay

Strains were grown overnight in PYE and diluted to an OD_660_ of 0.5 before inoculating 2 µL into PYE containing 0.3% agar. Plates were incubated at 30°C for 72 hours.

### Determining cell cycle phenotypes of CB15 populations

Strains were grown overnight in PYE, diluted to an OD_660_ of 0.05, and grown for 90 min. A volume of 2 µL of cells was immobilized on a 1% agarose pad. Microscopy was performed using a Nikon Ti-E inverted microscope equipped with an Orca Fusion BT digital CMOS camera (Hamamatsu). Fluorescence images were collected using a Prior Lumen 200 metal halide light source and a YFP- and mCherry-specific filter set (Chroma). Image analysis was performed with MicrobeJ (60).

### Live cell imaging of PleC-Venus and Venus-MipZ in unsynchronized cells

Strains were grown overnight in PYE, diluted to an OD_660_ of 0.05, and grown for 3 hours. A volume of 2 µL of cells was spotted onto a 1.5% agarose pad made with PYE and incubated at 30°C for 1 hour. Microscopy was performed with the same equipment described previously. Images were collected every 10 min for 3 hours.

### NA1000 synchronization

Strains were grown overnight in PYE, diluted to an OD_660_ of 0.1 in M2X, and grown for 6–8 hours. Cultures were diluted again into M2X and grown to an OD_660_ of 0.5–0.6. Cells were harvested and resuspended in chilled M2 salts and 1 volume of Percoll. Swarmer cells were separated by centrifugation at 7,000 × *g* for 20 min at 4°C. The bottom swarmer band was collected and washed with M2 salts.

### Overexpression and purification of FssC

A pET28a vector encoding 8xHis-SUMO-FssC was transformed into *E. coli* strain C43. Transformants were grown overnight and diluted (1/100) into 1 L of 2xYT media. Cultures were induced with 0.5 mM IPTG at an OD_600_ of 0.35 and incubated for 4 hours at 37°C. Cell pellets were stored at −80°C.

Cell pellets were resuspended in 30 mL lysis buffer (20 mM Tris-HCl pH 7.4, 1 M NaCl, 20 mM imidazole, 1 µM PMSF, and 10% glycerol) and passed through a cell disruptor at 20,000 psi. Lysates were centrifuged at 30,000 × *g* for 20 min at 4°C. Supernatant was supplemented with 0.1% PEI pH 7.25 and centrifuged again at 50,000 × *g* for 20 min at 4°C. A volume of 5 mL of Ni-NTA resin was added to the supernatant. The slurry was rocked for 1 hour at 4°C. The resin was washed with NWB (20 mM Tris-HCl pH 7.4, 300 mM NaCl, 10 mM imidazole, and 10% glycerol). Protein was eluted with NEB (20 mM Tris-HCl pH 7.4, 800 mM NaCl, 500 mM imidazole, and 10% glycerol). 6xHis-Ulp1 enzyme was added to the eluate and dialyzed against DC buffer (25 mM Tris-HCl pH 7.4, 300 mM NaCl, 10 mM imidazole, and 10% glycerol).

The cleavage reaction was transferred onto a column with 3 mL Ni-NTA resin. Flowthrough was concentrated with an Amicon Ultra 30000 MWCO. Concentrated sample was further purified with size exclusion chromatography using an AKTA Pure (GE Healthcare) FPLC system with a HiPrep 26/60 Sephacryl S-200 column. Fractions containing FssC were pooled, concentrated, and stored at −80°C.

### dNTP hydrolysis assays

Assays were performed in 50 mM Tris-HCl, pH 8, 100 mM NaCl, 0.4 mM DTT, and either 5 mM MnCl_2_ or MgCl_2_. All reactions contained 100 nM purified FssC and were incubated at 30°C. EDTA (65 mM) was added to quench reactions. FssC was precipitated with 1 volume of chilled methanol. Samples were analyzed by anion exchange using a DNAPac PA-100 (4 × 50 mm) column on a Shimadzu LC40 HPLC equipped with an SPD-M40 photodiode array detector. For reactions containing a single dNTP, the column was equilibrated with 25 mM Tris-HCl, pH 7.4 and 0.5 mM EDTA (buffer A). Injected sample (20 µL) was eluted with a 3 min isocratic phase of buffer A followed by a 10-min linear gradient of 0–500 mM LiCl. For reactions containing dGMP and/or multiple nucleotides, the column was equilibrated with 2.5% acetonitrile. Injected samples (25 µL) were eluted with a 25 min linear gradient of 0–175 mM potassium phosphate, pH 4.6. Absorbance was continuously monitored between 200 and 500 nm. Nucleotides were quantified by peak integration at 260 nm.

### Quantification of intracellular dNTPs

Nucleotides were extracted from cell cultures as described previously (61). Cultures were grown to an OD_660_ of 0.3–0.5 in M2X. Cells were harvested by vacuum filtration with a PTFE membrane (Satorius, SAT-11806-47-N). The membrane was submerged in chilled extraction solvent (50:50 [vol/vol] chloroform/water). Extracts were centrifuged to remove cell debris and the organic phase. The aqueous layer was stored at −80°C.

Samples were analyzed using an HPLC-tandem MS (HPLC-MS/MS) system with a Vanquish UHPLC system linked to heated electrospray ionization and a hybrid quadrupole high resolution mass spectrometer (Q-Exactive orbitrap, Thermo Scientific) operated in full-scan selected ion monitoring using negative mode. MS parameters included 70,000 resolution, 1e6 automatic gain control, 3.0 kV spray voltage, 40 ms maximum ion collection time, 35°C capillary temperature, and 70–1,000 *m/z* scan range. LC was performed on an Aquity UPLC BEH C18 column (1.7 µm, 2.1 × 100 mm; Waters). Injection volume was 25 µL. Flow rate was 0.2 mL/min, using Solvent A (97:3 [vol/vol] water/methanol, 10 mM tributylamine [Sigma-Aldrich] pH ~ 8.5 adjusted with acetic acid) and Solvent B (100% acetonitrile). The gradient was 95% A/5% B for 2.5 min, 90% A/10% B to 5% A/95% B over 14.5 min, then held for 2.5 min at 10% A/90% B. The gradient was returned to 95% A/5% B over 0.5 min and held for 5 min. HPLC eluate was sent to the MS for data collection from 3.3 to 18 min. Output data from the MS were converted to mzXML format using in-house-developed software. Metabolite quantification was performed by using the Metabolomics Analysis and Visualization Engine (MAVEN 2011.6.17, http://genomics-pubs.princeton.edu/mzroll/index.php) software suite. Peaks were matched to known standards for identification.

### qPCR to determine the ratio of *oriC/ter*

qPCR was performed on genomic DNA purified according to the Puregene DNA Handbook (Qiagen) protocol for gram-negative bacteria (62). Internal probes had 5′ fluorescein reporters and 3′ TAMRA quenchers. qPCR was performed with PrimeTime Gene Expression Master Mix (IDT), and 20 µL reactions were prepared according to the manufacturer’s directions in a MicroAmp optical 96-well plate. Reactions were conducted in a Quant Studio 7 Flex instrument with the following thermocycler program: 95°C for 3 min and 40 cycles of 95°C for 15 s and 60°C for 60 s. The average *C*_*T*_ value for technical replicates was used to calculate the relative copy number of *oriC* with the ∆∆C_T_ method.

### High-throughput sequencing to determine replication rates

Illumina sequencing libraries were prepared using the tagmentation-based and PCR-based Illumina DNA Prep kit and custom IDT 10 bp unique dual indices with a target insert size of 320 bp. Sequencing was performed on an Illumina NovaSeq 6000, producing 2 × 151 bp paired-end reads. Demultiplexing, quality control, and adapter trimming were performed with bcl-convert (version 4.1.5).

A total of 2.67 million reads were collected, mapped to the NA1000 genome with Bowtie2 (version 2.3.5.1), and sorted with Samtools (version 1.10). The number of reads/nucleotide position was determined with bedtools (version 2.27.1). Read counts were averaged over 5,000 bp windows and plotted as a function of chromosome position.

### Measuring intracellular c-di-GMP

Strains harboring pDH1397 integrated at the *xyl* locus were grown overnight in PYE, diluted into fresh PYE, and normalized by OD_660_. Fluorescence intensity was measured upon excitation/emission at 497/530 nm and normalized to the fluorescence intensity upon excitation/emission at 405/530 nm (47).

### Immunoblotting

SDS-PAGE was performed with 12% acrylamide gels and copolymerized with 35 µM Phos-tag (Nard) and 100 µM ZnCl_2_ when required. Proteins were transferred to a PVDF membrane in a semi-dry transfer apparatus (Trans-Blot Turbo, Biorad). Membranes were probed with a 1:10,000 dilution of anti-CtrA serum (63) or a 1:20,000 dilution of anti-DnaA (gifted by Paola E. Mera) and then incubated with a 1:10,000 dilution of goat anti-rabbit (H + L)-horseradish peroxidase (Biorad). Blots were developed with Western Lightning chemiluminescence substrate (Revvity) and imaged with an iBright 1500 imager (Invitrogen).

## Data Availability

Illumina sequencing data were uploaded to the Sequence Read Archive under BioProject PRJNA1096337.

## SUPPLEMENTAL MATERIAL

Supplemental material jb.00145-25-s0001.docx Supplemental methods, Tables S1 and S2, and Fig. S1 to S16.

ASM does not own the copyrights to Supplemental Material that may be linked to, or accessed through, an article. The authors have granted ASM a non-exclusive, world-wide license to publish the Supplemental Material files. Please contact the corresponding author directly for reuse.
