# Supplementary material for: A deoxynucleoside triphosphate triphosphohydrolase promotes cell cycle progression in Caulobacter crescentus
Source: J Bacteriol. 2025 Jun 2;207(6):e00145-25. doi: 10.1128/jb.00145-25 (PMC12186491; doi:10.1128/jb.00145-25)
Supplement: Supplemental material — Supplemental methods, Tables S1 and S2, and Fig. S1 to S16. [file jb.00145-25-s0001.docx]

**Supplemental Material**

A deoxynucleoside triphosphate triphosphohydrolase promotes cell cycle progression in *Caulobacter crescentus*.

Chandler N. Hellenbrand^a^, David M. Stevenson^a^, Katarzyna A. Gromek^a^, Daniel Amador-Noguez^a^, David M. Hershey^a^#

^a^ Department of Bacteriology, University of Wisconsin – Madison, Madison, WI 53706, USA

# To whom correspondence should be addressed: dhershey@wisc.edu

| **Contents of dNTP mix** | | | | **% dNTP Hydrolyzed** | | | |
| --- | --- | --- | --- | --- | --- | --- | --- |
| **dGTP** | **dATP** | **dCTP** | **dTTP** | **dGTP** | **dATP** | **dCTP** | **dTTP** |
| + | + | + | + | 97.81 | 12.72 | 78.50 | 45.59 |
| + | + | + | - | 94.12 | 3.59 | 58.16 | - |
| + | + | - | + | 97.72 | 10.70 | - | 37.25 |
| + | - | + | + | 2.33 | - | 1.20 | 1.61 |
| - | + | + | + | - | 1.06 | 48.09 | 23.21 |
| + | + | - | - | 95.96 | 2.50 | - | - |
| + | - | + | - | ND | - | ND | - |
| + | - | - | + | 2.02 | - | - | 1.62 |
| - | + | + | - | - | 2.08 | 52.01 | - |
| - | + | - | + | - | 7.04 | - | 39.24 |
| - | - | + | + | - | - | 2.40 | 2.10 |
| + | - | - | - | 5.14 | - | - | - |
| - | + | - | - | - | 4.47 | - | - |
| - | - | + | - | - | - | 0.97 | - |
| - | - | - | + | - | - | - | 1.68 |

**Table S1:** FssC hydrolysis only occurs in the presence of dATP when Mg^2+^ is the divalent cation. FssC enzyme was incubated with various mixtures of dNTPs (each totaling 500 µM) in reaction buffer containing Mg^2+^. The percent of each dNTP substrate hydrolyzed after 4 hours is shown. Substantial hydrolysis only occurs when dATP is present with other dNTPs. ND = no hydrolysis detected.

| **Fig. 1D** |  |  |  |
| --- | --- | --- | --- |
| **Strain** | **Cell Type** |  |  |
|  | Swarmer | Stalked | Predivisional |
| WT | 0.077673 | 0.046085 | 0.039152 |
| ∆*fssC* | 0.036339 | 0.013275 | 0.026642 |
| **Fig S5C** |  |  |  |
| **Strain** | **Cell Type** |  |  |
|  | Swarmer | Stalked | Predivisional |
| ∆*fssC* *xyl::P_fssC_*-empty | 0.002021 | 0.027515 | 0.025744 |
| ∆*fssC* *xyl::P_fssC_*-*fssC* | 0.097206 | 0.035492 | 0.063769 |
| ∆*fssC* *xyl::P_fssC_*-*fssC* H102AD103A | 0.058236 | 0.068206 | 0.014625 |

**Table S2:** Standard deviations for data in Figure 1D and Figure S6C.


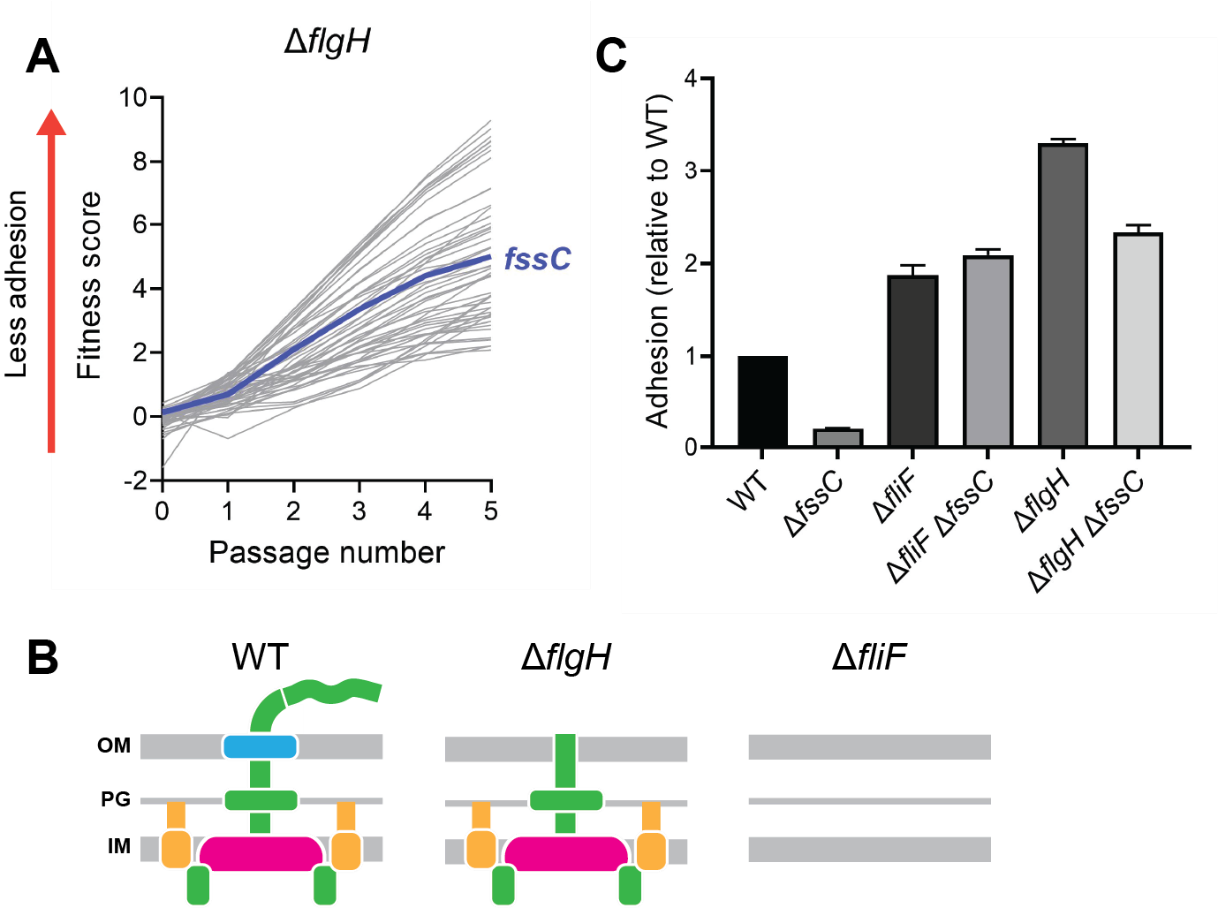


**Figure S1:** *fssC* contributes to adhesion when the surface sensing pathway is activated. A) A transposon library in the ∆*flgH* genetic background was passaged through cheesecloth to identify genes that contribute to surface adhesion downstream of the surface sensing pathway. *fssC* (highlighted in blue) was identified in this screen. B) Structure of the flagellum in WT, ∆*flgH*, and ∆*fliF* backgrounds. *flgH* codes for the L-ring (blue) in the outer membrane. *fliF* codes for the MS ring (pink) in the inner membrane. Deletion of *flgH* causes the synthesis of an incomplete flagellum that constitutively activates the surface sensing pathway through the stators (yellow). The ∆*fliF* mutant cannot activate this pathway because the stators cannot assemble with the rest of the rotor complex. C) Adhesion of *C. crescentus* CB15 strains measured by a crystal violet-based biofilm assay in M2X. ∆*flgH* is hyper-adhesive compared to WT because the signaling pathways that lead to adhesion after surface contact are constitutively activated. *fssC* only contributes to adhesion in backgrounds that can activate the surface sensing pathway. Error bars represent the standard deviation of the mean for 3 biological replicates.


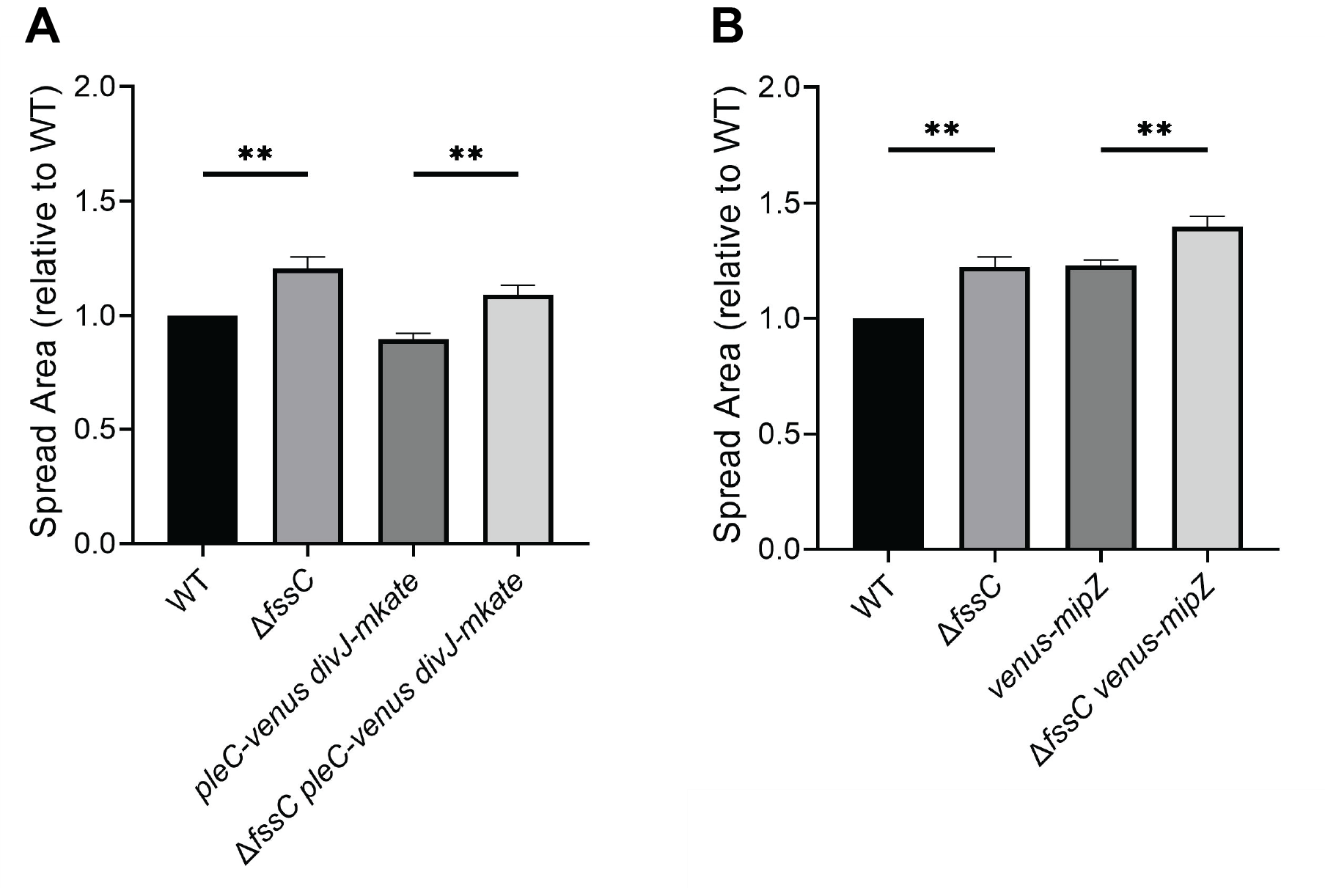


**Figure S2:** The ∆*fssC* soft agar phenotype is maintained in reporter strains with pleC*-venus, divJ-mkate,* and *venus-mipZ* alleles. A) The *pleC-venus divJ-mkate* CB15 reporter strains do not spread as far through the soft agar, but the difference between WT and ∆*fssC* is proportional to the difference in the WT background. B) The *venus-mipZ* NA1000 reporter strain spreads slightly farther through the soft agar, but the difference between WT and ∆*fssC* is proportional to the difference in the WT background. Error bars represent the standard deviation of the mean for 3 biological replicates. Statistical comparisons were made with an unpaired t-test. ***P* < 0.01.


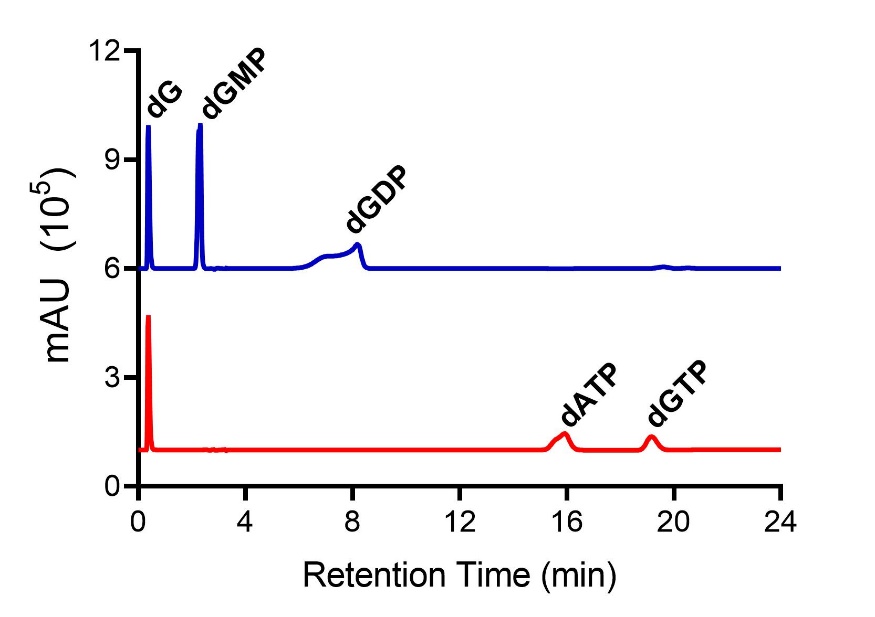


**Figure S3:** FssC hydrolyzes dGTP into deoxyguanosine (dG). FssC enzyme was incubated with 500 µM dGTP in reaction buffer supplemented with Mg^2+^ and 250 µM dATP for 4 hours. There is minimal dATP hydrolysis (~4%) under these conditions. Contents of the reaction (red) were separated with an anion exchange method optimized to separate possible nucleotide products. A standard of dG, dGMP, and dGDP (blue) was used to identify the product of FssC hydrolysis. These chromatograms demonstrate that FssC is a triphosphohydrolase and converts dGTP into dG.


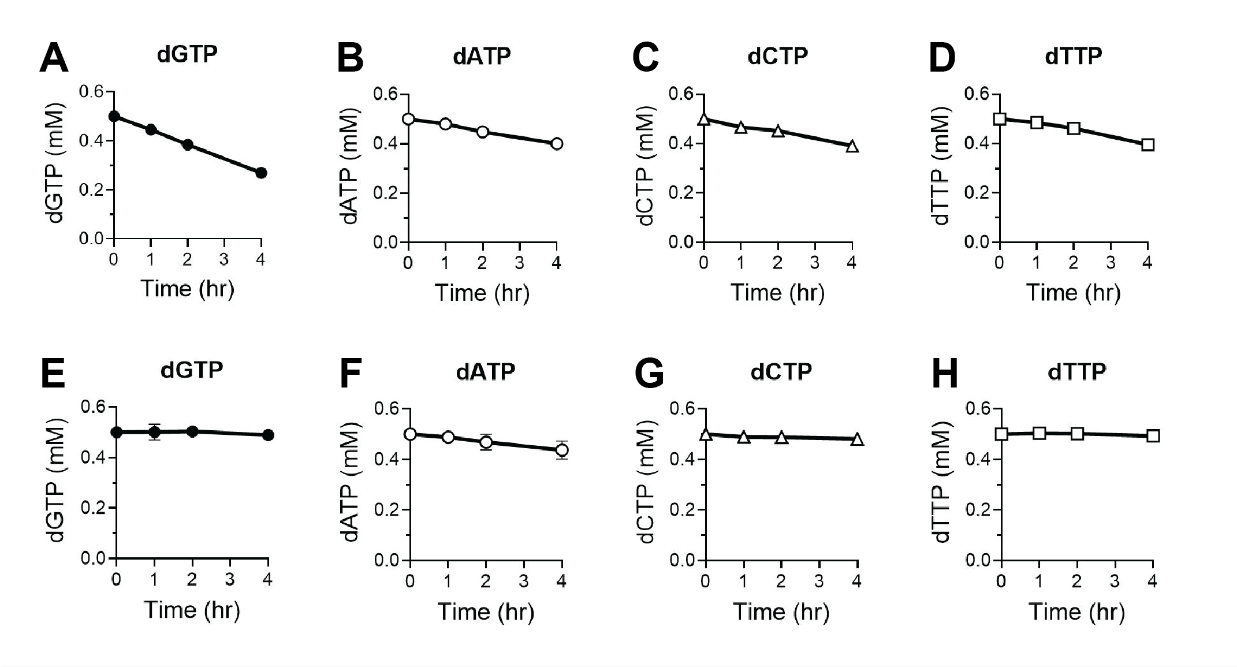


**Figure S4:** *In vitro* hydrolysis activity of FssC towards individual dNTPs in reaction buffer containing Mn^2+^ (A-D) or Mg^2+^ (E-H). Graphs show the concentration of substrate remaining after incubating with FssC enzyme for 1, 2, and 4 hrs. FssC has a kinetic preference for dGTP over the other dNTPs when the reaction buffer is supplemented with Mn^2+^. However, FssC does not hydrolyze individual dNTPs when the reaction buffer is supplemented with Mg^2+^ (see Table S1). Error bars represent the standard deviation of the mean for 3 replicates.


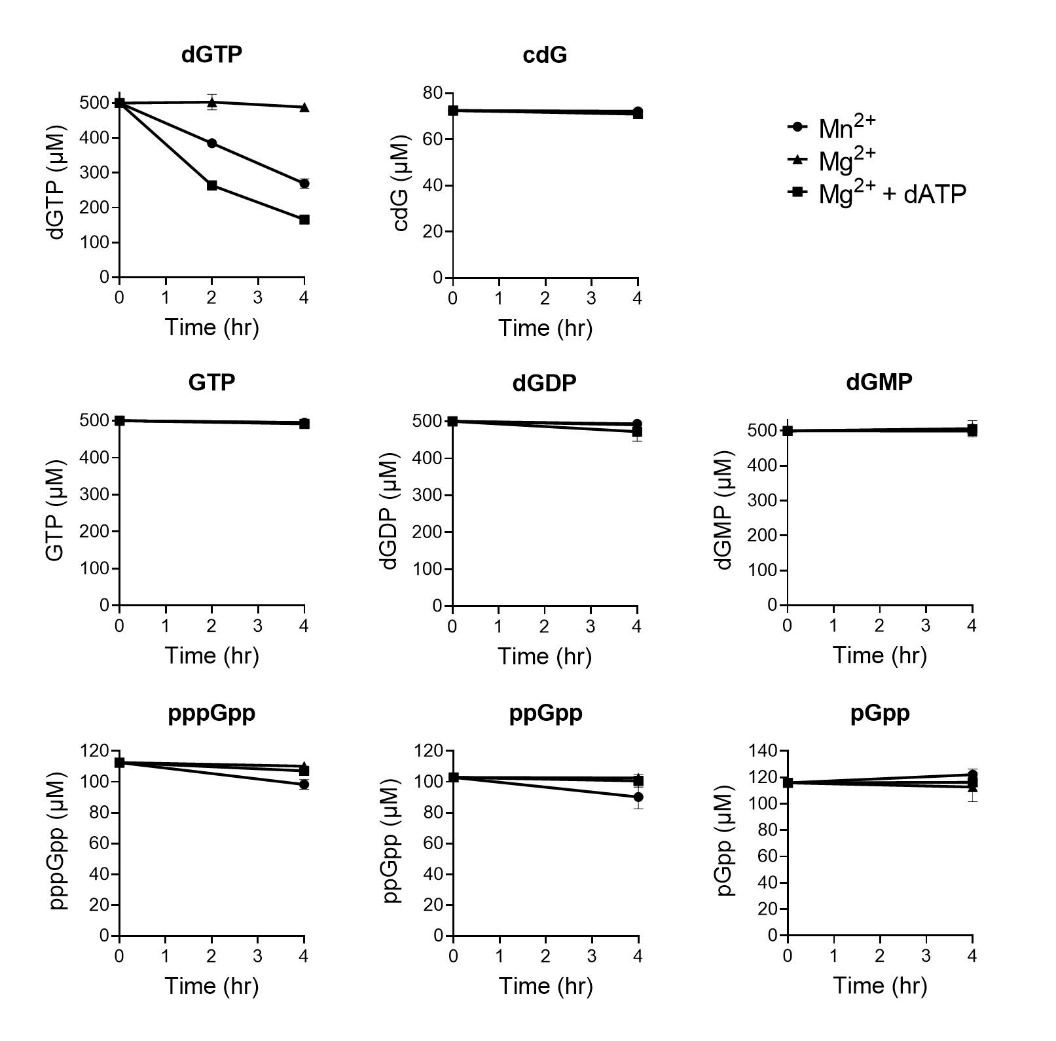


**Figure S5:** FssC does not hydrolyze nucleotides other than dNTPs *in vitro*. Assays were performed under 3 conditions: with Mn^2+^ as the cation in the reaction buffer, with Mg^2+^ as the cation in the reaction buffer, and with Mg^2+^ plus the addition of 250 µM dATP (FssC activating conditions, see Table S1). Error bars represent the standard deviation of the mean for 3 replicates. Data is normalized to a control reaction with no enzyme present. None of the nucleotide substrates tested (GTP, c-di-GMP (cdG), dGDP, dGMP, pppGpp, ppGpp, pGpp) exhibited hydrolysis by FssC. There appears to be some hydrolysis of pppGpp and ppGpp in the Mn^2+^ buffer, but this is likely due to the instability of these nucleotides under this condition. pppGpp and ppGpp hydrolysis reached 50-60% after 4 hours in control reactions without added enzyme, and therefore the slight hydrolysis seen here is more likely from substrate instability than from hydrolysis by FssC.


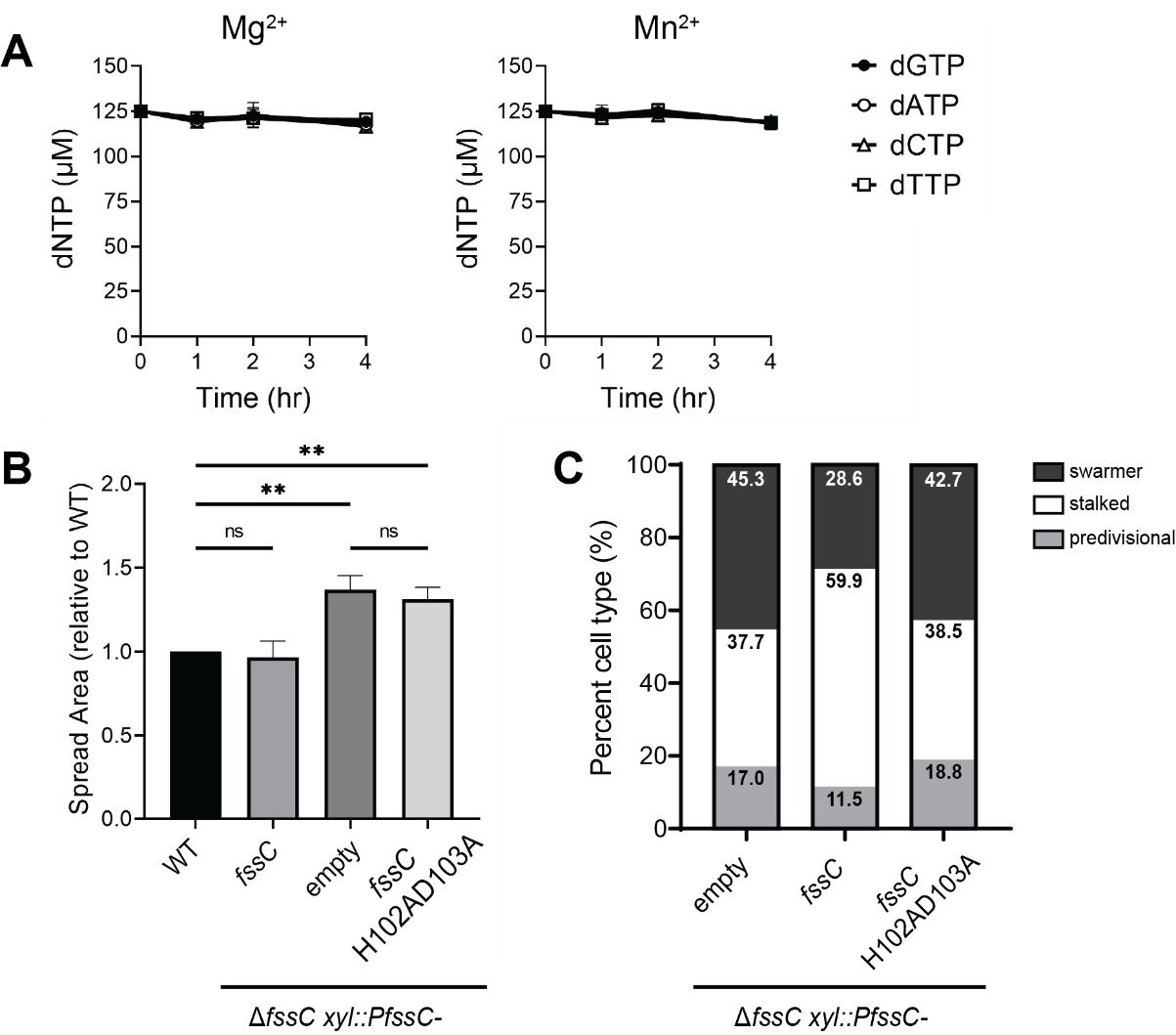


**Figure S6:** The FssC H102A D103A variant does not hydrolyze dNTPs or complement ∆*fssC*. A) The H102A D103A mutant is catalytically inactive *in vitro* and does not hydrolyze dNTPs with either Mg^2+^ or Mn^2+^. Error bars represent the standard deviation of the mean for three replicates. B) Wild-type and H102A D103A *fssC* alleles were ectopically expressed at the *xyl* locus under the native *fssC* promoter. Expression of the wild-type *fssC* allele reverses the hyper-spreading phenotype of the ∆*fssC* mutant on soft agar, while expression of the catalytically inactive mutant does not. Error bars represent the standard deviation of the mean for 3 biological replicates. C) Expression of the *fssC* H102A D103A variant does not decrease the percentage of swarmer cells in unsynchronized ∆*fssC* populations (*P* = 0.5583), but expression of the WT allele does (*P* = 0.0006). Each bar represents n > 800 cells collected over 3 biological replicates. Standard deviations can be found in Table S2. All statistical comparisons were made using an unpaired t-test. ***P* < 0.01.


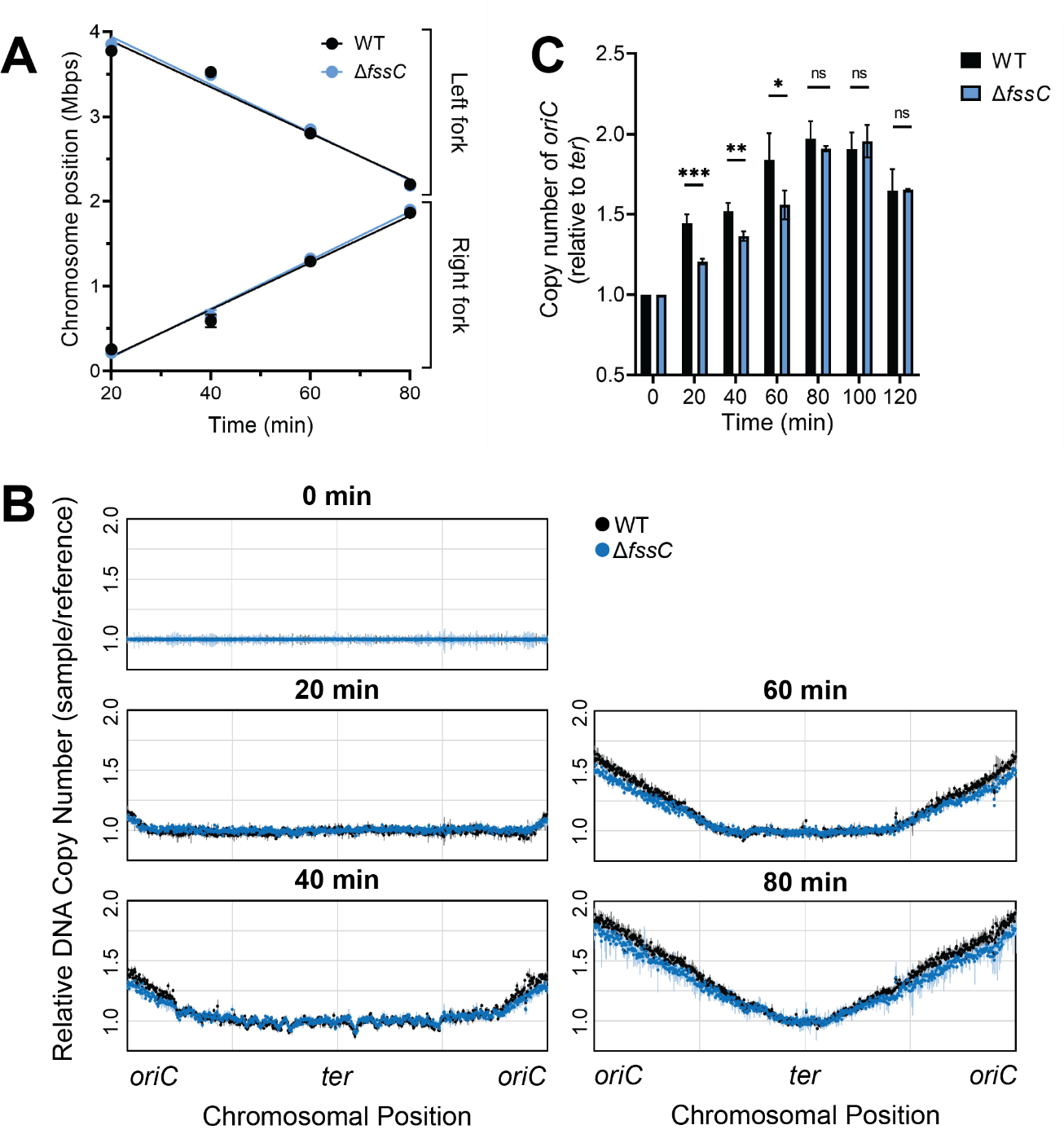


**Figure S7:** Replication rate and relative copy number of *oriC* in M2X medium. A) Positions of the right and left replication forks are plotted as a function of time for WT (black) and ∆*fssC* (blue). The line of best fit is shown for 3 biological replicates. Slopes are not significantly different (*P* = 0.6094 for right forks, *P* = 0.5601 for left forks). P-values were determined by an extra sum-of-squares F-test. The left and right forks in WT had replication rates of 452 ± 27 and 460 ± 22 bp/s, and the left and right forks in ∆*fssC* had rates of 470 ± 21 and 477 ± 16 bp/s, respectively. B) Replication was monitored in synchronized NA1000 cells with a high-throughput sequencing approach. Read counts for each chromosomal position were normalized to t=0 to calculate relative copy number across the chromosome. Replication forks (black arrows) are at the interface between replicated and unreplicated DNA. The average of 3 biological replicates is shown; error bars represent the standard deviation of the mean. C) Relative copy number of *oriC* in synchronized populations over time as determined by qPCR. Primers were designed for *oriC* and the *ter* region (see Fig. 3A). The amount of *oriC* in each sample was normalized to the amount of *ter* and then to t=0. Error bars represent the standard deviation of the mean for 3 biological replicates. Statistical comparisons were made using an unpaired t-test. **P* < 0.05, ***P* < 0.01, ****P* < 0.001.


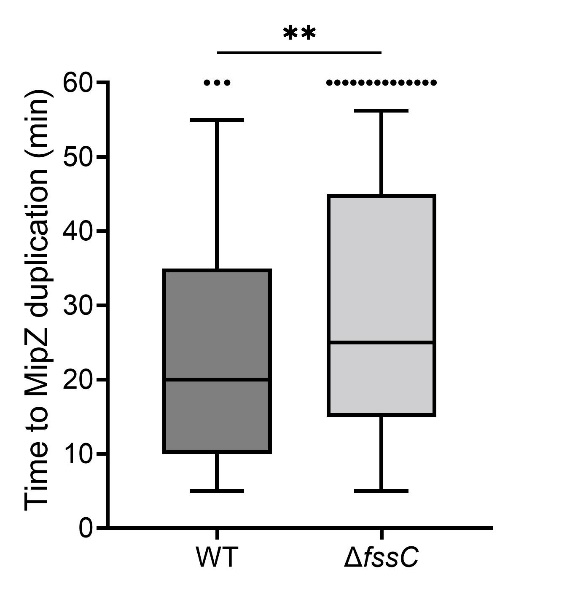


**Figure S8:** Venus-MipZ duplication in synchronized NA1000 cells. ∆*fssC* has on average a 5-minute delay in MipZ duplication compared to WT. Box and whisker plots show the 5-95 percentile. Data is compiled from n=158 WT cells and n=294 ∆*fssC* cells. Statistical comparisons were made using an unpaired t-test. ***P* < 0.01.


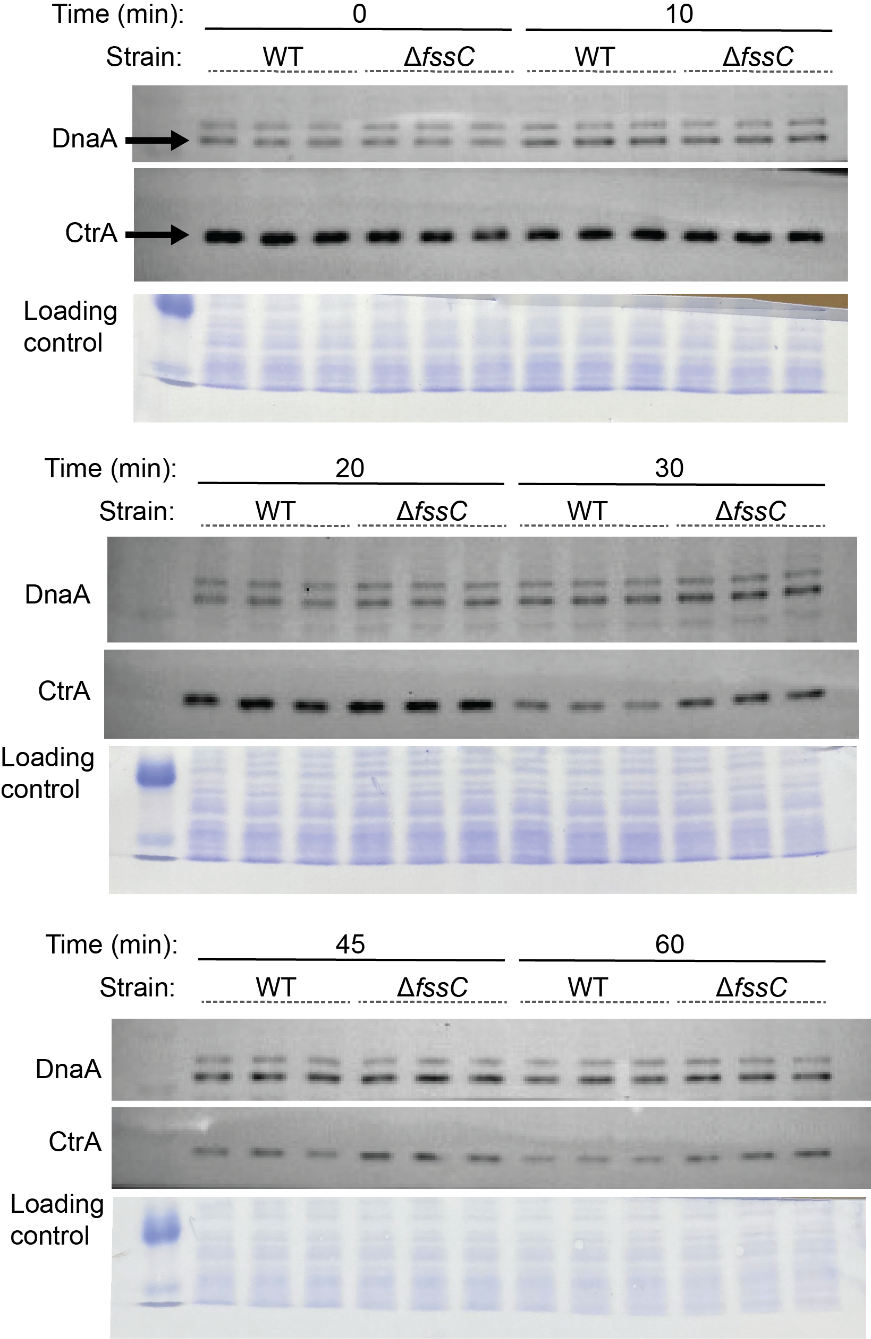


**Figure S9:** Immunoblots corresponding to Figure 5C.


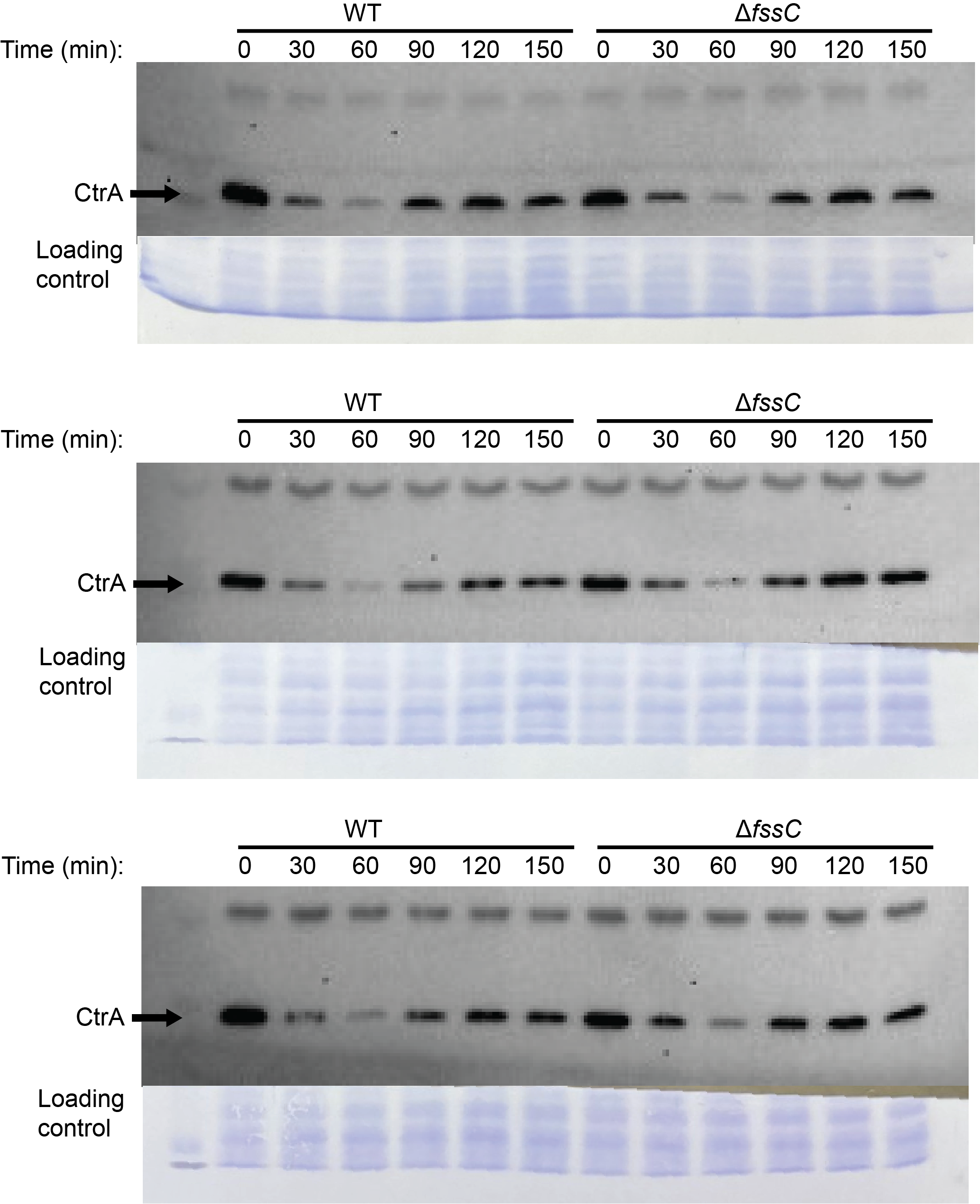


**Figure S10:** Immunoblots corresponding to Figure 6A.


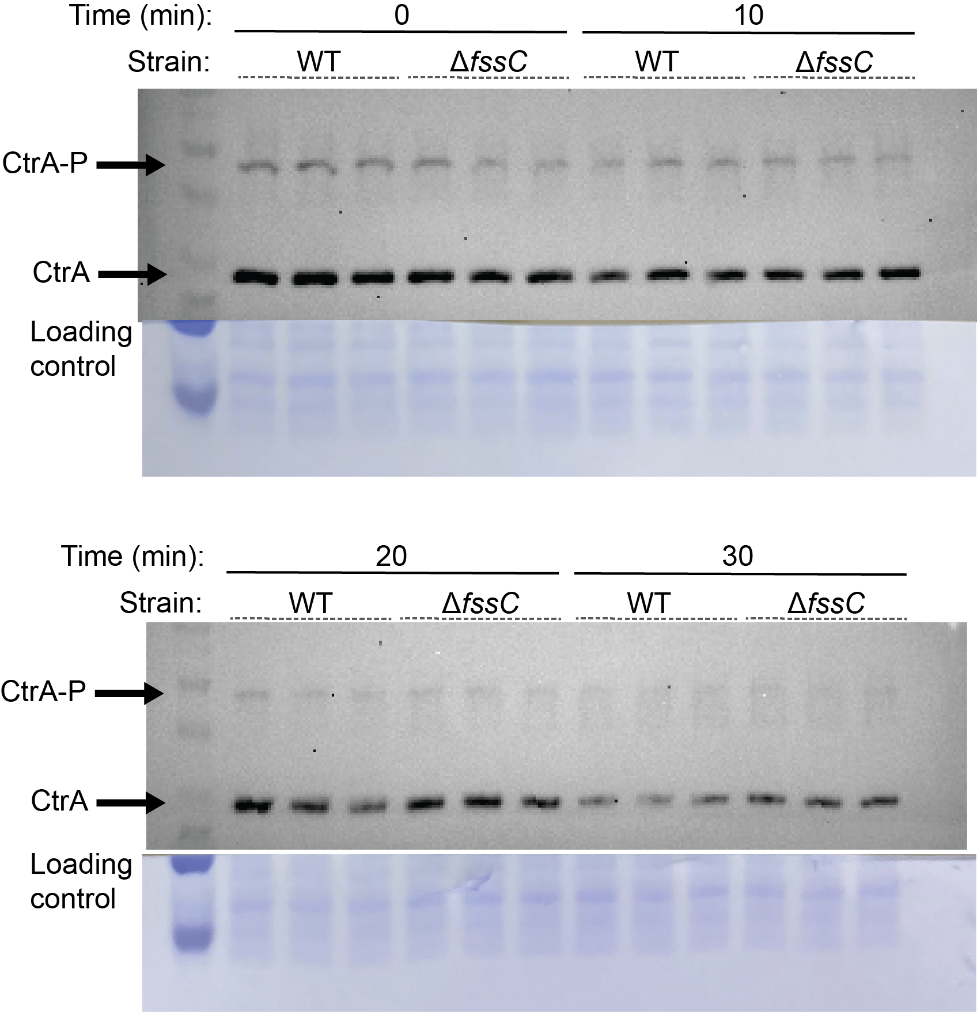


**Figure S11:** Immunoblots corresponding to Figure 6C.


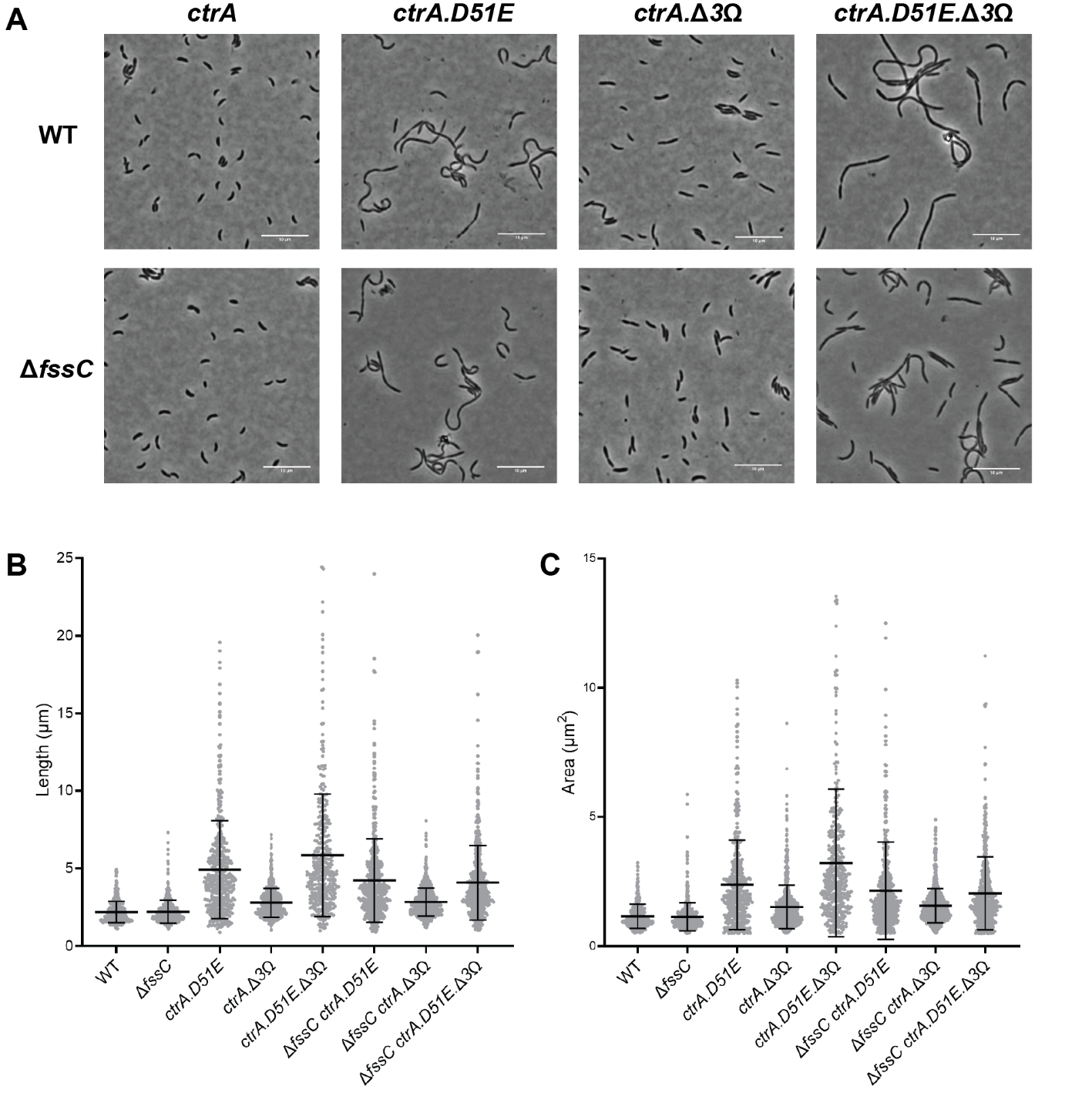


**Figure S12:** ∆*fssC* cells have the same length and area as WT cells. A) Micrographs showing the cell morphology of phosphomimetic (*ctrA.D51E*) and stabilized (*ctrA.*∆*3*Ω*) ctrA* alleles in the WT and ∆*fssC* backgrounds. Cells harboring the *ctrA.D51E* allele have a filamentous morphology not previously reported in the literature, likely because our *ctrA* variants are expressed as the only copy of *ctrA* from the native locus and not from a high copy number plasmid (Domian et al., 1997). Scale bars are 10 µM. Cell shape analysis was performed on each strain to measure the length (B) and area (C) for n > 350 cells. The ∆*fssC* mutant has the same length and area as WT (*P* = 0.6647 and *P* = 0.5095, respectively), and does not have the filamentous morphology of the *ctrA.*∆*3*Ω variant (*P* < 0.0001 for length and area of *ctrA.*∆*3*Ω cells compared to WT). Error bars show the standard deviation of the mean, and statistical comparisons were made using an unpaired t-test.

**
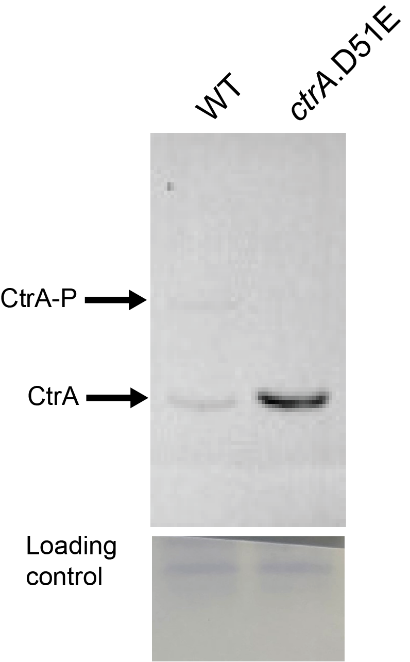
**

**Figure S13:** The CtrA.D51E phosphomimetic variant is not phosphorylated. The band corresponding to phosphorylated CtrA is not present when *ctrA.D51E* lysates are run on a Phos-Tag™ polyacrylamide gel.


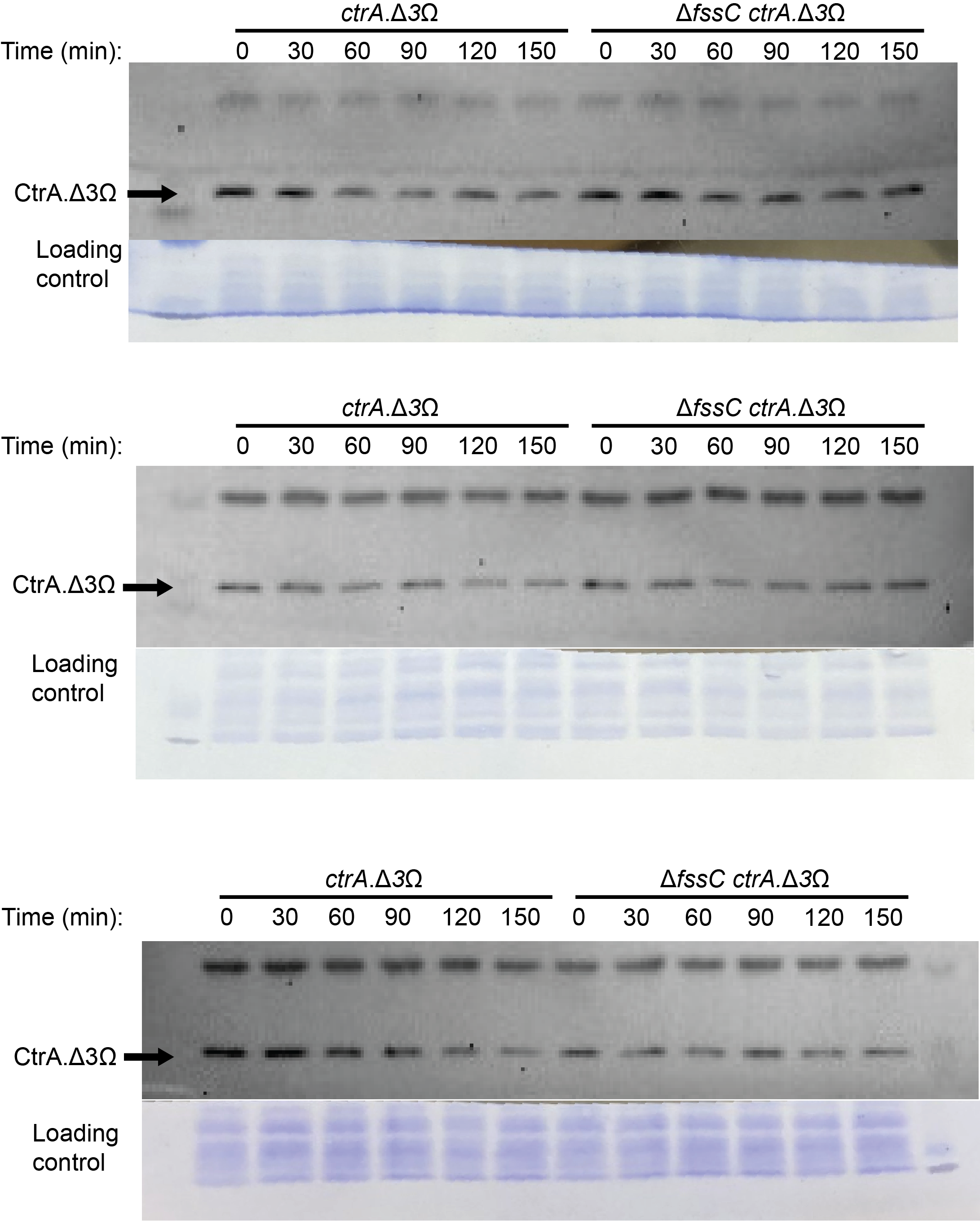


**Figure S14:** Immunoblots corresponding to Figure 6B.


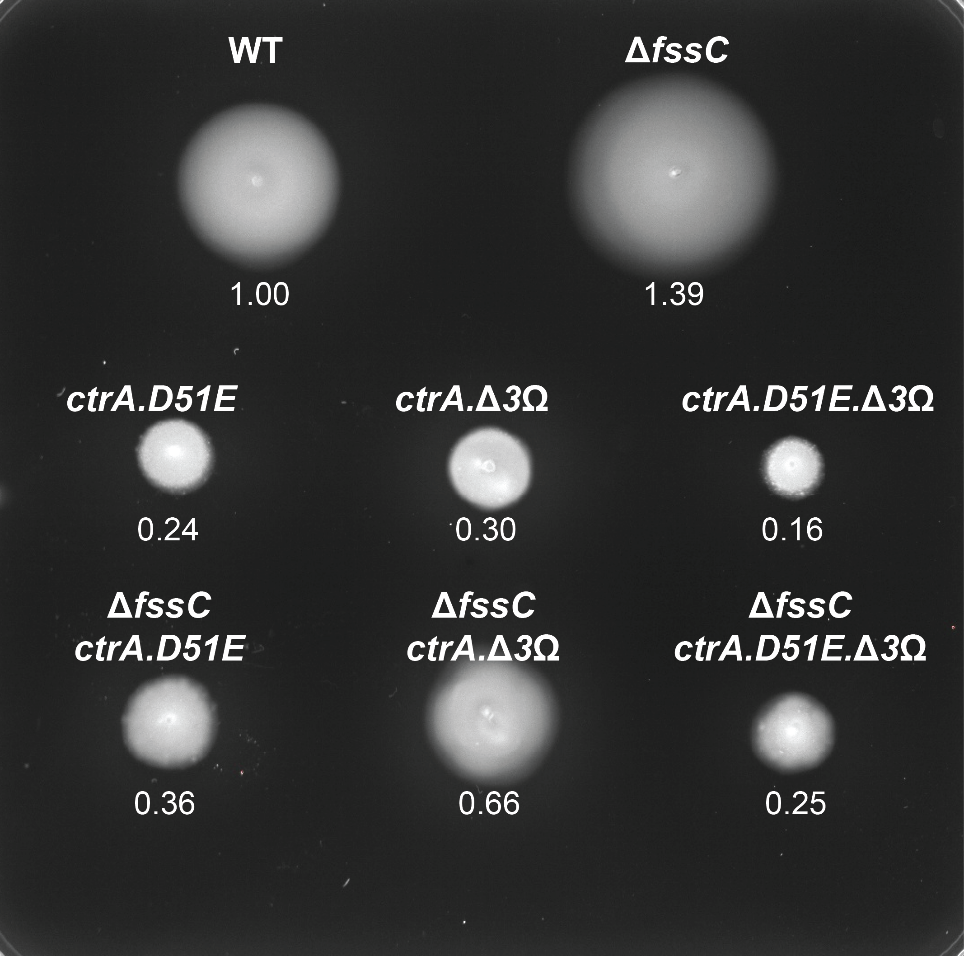


**Figure S15:** Representative soft agar plate from Figure 6D.


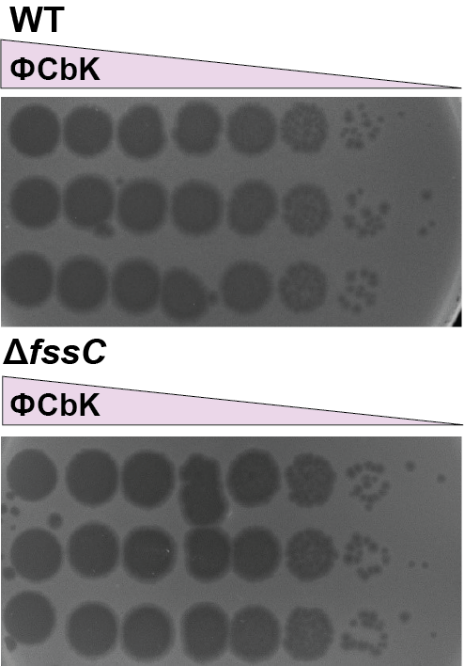


**Figure S16:** *fssC* does not influence susceptibility to ɸCbK infection. ɸCbK is a caulophage that transfects through the pilus portals. A serial dilution plaque assay demonstrated that WT and the ∆*fssC* mutant are equally sensitive to ɸCbK, indicating that *fssC* is not involved in phage defense.

**Supplemental Methods**

*Quantification of surface adhesion by crystal violet staining*

Overnight cultures were grown in PYE with 4 biological replicates. Cultures were diluted to an OD_660_ of 0.5 before 2 µL were inoculated in 450 µL M2X in a 48 well plate and grown at 30°C shaking. After 18 hrs, cultures were dumped, and plates were washed with tap water. Cells attached to the plate were stained by the addition of 500 µL 0.01% crystal violet. After incubating at room temperature for 10 min shaking, the dye was dumped, and plates were washed again with tap water. Remaining crystal violet was dissolved with 500 µL ethanol. After shaking for another 10 min, absorbances were read at 575 nm.

*Live cell imaging of Venus-MipZ in synchronized cells*

NA1000 strains with *venus*-*mipZ* were synchronized in the swarmer phase as described above. Isolated swarmers were resuspended in PYE to an OD_660_ of 0.25, and 2 µL of cells were spotted onto a 1% agarose pad made with PYE medium. Microscopy was performed using a Nikon Ti-E inverted microscope equipped with an Orca Fusion BT digital CMOS camera (Hamamatsu). Fluorescence images were collected using a Prior Lumen 200 metal halide light source and a YFP-specific filter set (Chroma). Time-lapse images were taken every 5 min for 60 min, and image analysis was performed with MicrobeJ.

*Plaque Assays*

PYE top agar containing 0.3% agar was melted and cooled to room temperature. 100 µL of saturated WT or ∆*fssC* culture was added to 5 mL of top agar and spread evenly on a PYE plate. Phage ɸCbK was serially diluted in PYE from 10^0^ to 10^-7^. 3 µL of each dilution was spotted onto the solidified top agar. Plates were sealed with parafilm and incubated at 30°C overnight.
